# miR-200c inhibition and catalase accelerate diabetic wound healing

**DOI:** 10.1186/s12929-024-01113-7

**Published:** 2025-02-14

**Authors:** Marco D’Agostino, Sara Sileno, Daniela Lulli, Naomi De Luca, Claudia Scarponi, Massimo Teson, Alessio Torcinaro, Francesca De Santa, Corrado Cirielli, Sergio Furgiuele, Chris H. Morrell, Elena Dellambra, Teresa Odorisio, Edward G. Lakatta, Daniele Avitabile, M. C. Capogrossi, Alessandra Magenta

**Affiliations:** 1 Molecular Regenerative Medicine Laboratory, Istituto Dermopatico dell’Immacolata (IDI-IRCCS), Rome, Italy; 2 Experimental Immunology Laboratory, Istituto Dermopatico dell’Immacolata (IDI-IRCCS), Rome, Italy; 3 Laboratory of Molecular Biology, Istituto Dermopatico dell’Immacolata (IDI-IRCCS), Rome, Italy; 4 Institute of Biochemistry and Cell Biology (IBBC), National Research Council of Italy (CNR), Rome, Italy; 5 Unit of Vascular Surgery, Istituto Dermopatico dell’Immacolata (IDI-IRCCS), Rome, Italy; 6 Unit of Vascular and Endovascular Surgery, High Speciality Hospital “Mediterranea”, Naples, Italy; 7 Laboratory of Cardiovascular Science, National Institute on Aging (NIA), National Institutes of Health (NIH), Baltimore, MD USA; 8 Idi Farmaceutici S.r.l., Pomezia, Italy; 9 Division of Cardiology, Johns Hopkins Bayview Medical Center, Johns Hopkins University, Baltimore, MD USA; 10 Institute of Translational Pharmacology (IFT), National Research Council of Italy (CNR), Rome, Italy

**Keywords:** Diabetic foot ulcers, MicroRNAs, Catalase, Skin, Reactive oxygen species, Apoptosis

## Abstract

**Background:**

Reactive oxygen species (ROS) are increased in diabetic conditions and play a causal role in diabetic foot ulcers (DFU). We previously showed that ROS up-regulate miR-200c expression, that in turns causes apoptosis, senescence, ROS upregulation and nitric oxide decrease, leading to endothelial disfunction.

**Methods:**

The aim of this study is to dissect miR-200c role in DFU and to explore the potential role of anti-miR-200c and antioxidant catalase (CAT) in promoting wound healing (WH). miR-200c inhibition and CAT treatment were performed either in immortalized keratinocytes (HaCaT) or in primary fibroblasts (FBs) and keratinocytes (KCs) deriving from diabetic patients (pts) undergoing amputations. Primary cells deriving from pts undergoing saphenectomies were used as controls. The miR-200c blockade was performed either via lentiviral particles bearing an anti-miR-200c sequence or locked nucleic acid (LNA) anti-miR-200c oligos. Equine CAT was administered on cell medium. The WH assay was performed in vivo on diabetic (db/db) mice by a topical treatment with CAT and LNA anti-miR-200c on wounds dissolved in a Pluronic gel mixture, administered every three days.

**Results:**

We found that miR-200c levels were increased by different stimuli known to induce ROS, such as ultraviolet radiation (UV), hydrogen peroxide (H_2_O_2_), and high glucose in HaCaT. miR-200c was also upregulated in skin biopsies, in FBs and KCs isolated from pts with DFU *vs* controls. Forced miR-200c expression induced ROS in both FBs and KCs, and CAT reduced it. miR-200c inhibition improved WH in HaCaT, both under basal conditions and after UV and H_2_O_2_ treatment, and the simultaneous treatment with CAT accelerated it. miR-200c inhibition accelerated WH in KCs of DFU pts, increasing its protein targets: sirtuin 1 (SIRT1), the transcription factors FOXO1 and ZEB1 and decreasing p66Shc phosphorylation at Ser-36, that is induced by ROS, and the co-treatment with CAT showed synergistic effects in reducing ROS and cytotoxicity. Interestingly, CAT treatment decreased miR-200c expression in FBs and KCs of DFU pts. Topical administration of anti-miR-200c and CAT in a WH model of diabetic mice accelerated closure.

**Conclusions:**

Anti-miR-200c and CAT could be considered a novel treatment for DFU and, possibly, for other types of non-diabetic skin ulcers.

**Supplementary Information:**

The online version contains supplementary material available at 10.1186/s12929-024-01113-7.

## Background

Diabetes Mellitus (DM) is characterized by increased reactive oxygen species (ROS), inflammation, thrombosis, and atherogenesis [1] leading to foot ulcers, a major concern in DM patients (pts), necessitating urgent attention [2].

microRNA (miRNAs) are small non-coding RNAs (about 22 nt) that regulate stability/or translation efficiency of messenger RNA (mRNA), that are deregulated in different pathologic conditions [3].

We showed that the miR-200 family is upregulated by ROS in various cell types including endothelial (ECs), fibroblasts (FBs), and muscle cells [4–6] and in ROS-associated pathophysiological conditions including ischemia, atherosclerosis, psoriasis, familial hypercholesterolemia, Duchenne muscle dystrophy and aging [4–10]. The most up-regulated member is miR-200c, that is responsible of apoptosis, senescence and inflammation via reducing the transcription factor zinc finger E-box binding homeobox 1 (ZEB1) [7, 11]. Furthermore, miR-200c targets Sirtuin 1 (SIRT1), an anti-inflammatory, antiapoptotic and antioxidant protein, and Forkhead box protein O1 (FOXO1), a transcription factor that transcribes antioxidant genes, such as catalase (CAT) and manganese superoxide dismutase (MnSOD), and SIRT1 [5]. miR-200c also targets endothelial nitric oxide synthetase (eNOS) [5], reducing nitric oxide (NO), that is crucial for wound healing (WH) and also to stabilise the mRNA and protein of SIRT1 [12]. Moreover, miR-200c up-regulates ROS causing the phosphorylation at Ser-36 of the oxidative stress sensor p66Shc protein (p66Shc P-Ser36), that further enhances ROS in different cell compartments, contributing to endothelial dysfunction [5]. p66Shc is a redox enzyme implicated in the translation of oxidative signals [13], the phosphorylation of the Ser-36 site is crucial for oxidative stress response [14, 15]. p66Shc modulates ROS production by three mechanisms localized in the nucleus, plasma membrane, and mitochondria. The nuclear mechanism is mediated by the inhibition of FOXO transcription factors, leading to the decreased expression of ROS-scavenging enzymes CAT and MnSOD [16]. At the plasma membrane, increases ROS production via the NADPH membrane oxidase pathway. Finally, p66Shc induces apoptosis, as phosphorylation at Ser-36 is followed by isomerization by the peptidyl-prolyl cis/trans isomerase PIN1. This isomerization enables the dephosphorylation of the Ser-36 residue by the serine/threonine phosphatase PP2A, facilitating its translocation from the cytosol to the mitochondrial intermembrane space (IMS). In the IMS, p66Shc binds to cytochrome c, functioning as an oxidoreductase that generates ROS, triggering the release of mitochondrial apoptotic factors and ultimately inducing cell apoptosis [17]. In addition, its phosphorylation is mediated by the ROS-induced PKCβII activation. Interestingly, the mitochondrial H_2_O_2_ production induced by p66Shc, further increases intracellular H_2_O_2_ levels, which in turn enhance PKCβII activation in a positive control loop [18, 19]. In agreement with a p66Shc proapoptotic role, it can be also phosphorylated by other kinases such as: p38 MAPK [20] and the apoptosis signal-regulating kinase 1 (ASK1) [21]. p38 MAPK is activated by different stimuli that induce ROS, such as thermic and osmotic shock, inflammatory cytokines, lipopolysaccharides, ultraviolet light, IL-1, and H_2_O_2_ [20]. Indeed, p66Shc knockout mice (p66Shc − / −) show increased resistance to oxidative stress, reduced oxidative stress-induced apoptosis, prolonged lifespan, decreased production of intracellular oxidants, and increased resistance to oxidative stress-induced apoptosis, which can be restored by p66Shc overexpression [22].

Since both ischemia and hyperglycaemia cause ROS upregulation, we investigated whether miR-200c was involved in delayed WH in diabetic foot ulcers (DFU). Additionally, the antioxidant enzyme CAT, which is known to ameliorate WH [23–25] and used in in topical pharmaceutical preparations to accelerate wound repair [26, 27], was included in the study. This study aims to evaluate the expression of miR-200 family members and relevant targets in DFU biopsies, keratinocytes (KCs), and fibroblasts (FBs), and to assess the effects of anti-miR-200c alone and in combination with CAT in WH assays in vitro and in diabetic mice in vivo.

## Methods

### Subject selection and classification

Pts affected by type 2 diabetes (DM) and DFU (DB, N = 10) undergoing limb amputation and normoglycemic healthy subjects without skin ulcer undergoing saphenectomy (HS, N = 8) were prospectively enrolled from 2020 to 2022 at the Vascular Surgery Unit of IDI-IRCCS, Rome, Italy. Clinical parameters were also recorded. In supplementary Table 1 are described clinical and laboratory parameters of DFU pts: age, sex, fasting glucose, haemoglobin glycated (HbA1C) and duration of diabetes.

### Skin samples

Skin samples were collected from 10 DB pts and 8 HS following the standard laboratory procedures. In all DB pts, 6 mm punch biopsies were taken from skin of normal-appearing non-lesional skin (NLS), distant from sites of wound lesions. Skin biopsies were collected in MACS tissue storage solution (Miltenyi Biotech, Auburn, CA, USA) and immediately frozen in nitrogen liquid at – 80 °C or embedded in paraffin or in Optimal cutting temperature compound (OCT) [28] (Killik-O.C.T. Compound neutral, Bio-Optica Milano S.p.A., Milan, Italy) for cryosections.

### Cell culture

KCs were established from biopsies obtained from NLS of DB pts undergoing major amputation. Control KCs were extracted from HS undergoing saphenectomies. The specimens were collected by the vascular surgery unit of IDI-IRCCS. KCs were cultured on a feeder layer of lethally irradiated 3T3–J2 cells, according to the method described by Rheinwald and Green [29]. Briefly, 2 cm^2^ skin biopsies were minced and trypsinized (0.05% trypsin/0.01% EDTA) at 37 °C for 3 h. Afterward, cells were collected every 30 min, plated on lethally irradiated 3T3–J2 cells, and cultured in 5% CO_2_ in KC growth medium (KGM): Dulbecco–Vogt Eagle’s and Ham’s F12 media (3:1 mixture) containing 10% FCS, insulin (5 µg/ml), transferrin (5 µg /ml), adenine (0.18 mM), hydrocortisone (0.4 µg/ml), cholera toxin (0.1 nM), triiodothyronine (20 pM), epidermal growth factor (EGF) (10 ng/ml), and penicillin/streptomycin (50 IU/ml). Confluent primary cultures were trypsinized and were plated in secondary cultures. Second or third passage cultured KCs were used in all experiments, with cells cultured in the serum-free medium KGM (Clonetics, San Diego, CA, USA) for at least 3–5 days (about 70% confluence) before performing treatments. FBs were established from NLS of DB pts and HS undergoing plastic surgery. Briefly, full-thickness skin biopsies were cleaned with surgical scissors to remove most of the subcutaneous tissue and the remaining skin was finely minced into small pieces. These pieces were placed in flasks and cultured in Dulbecco’s modified Eagle’s medium (DMEM) containing 10% foetal bovine serum (FBS), 50 IU–50 µg/ml penicillin–streptomycin and 4 mM glutamine) to obtain human fibroblasts (FBs). Primary FB cultures were amplified two times and subconfluent third-passage FB cultures were used for further molecular experiments. Immortalized keratinocytes (HaCaT) cells were grown in DMEM supplemented with 10% FBS (Euroclone S.p.A., MI, Italy).

Bone marrow (BM) cells were isolated from hindlimbs (femurs and tibiae) of male and female C57-BL6 mice and, after lysis of red blood cells were plated in BM medium (high-glucose DMEM supplemented with Glutamax, Pen/Strep, 20% FBS, 30% L929-conditioned medium, 0.5% sodium pyruvate, 0.1% β-mercaptoethanol). Cultures were fed by adding fresh medium every two days. Stimulations with lipopolysaccharides (LPS) (10 ng/ml) and interleukin (IL)−4 (20 ng/ml) were carried out at day 6.

### Cell treatments and inflammatory stimulation

Subconfluent HaCaT were treated with 50 J/m^2^ UV of 254 nm, that is in the range of UVC (UV Stratalinker 1800, STRATAGENE, CA, USA), in absence of media, then complete media were added, and cell were collected after 16 h (h). In scratch assay experiments serum free media were added after UV treatments in presence or not of CAT for 72 h. HaCaT were treated with 400 μM H_2_O_2 _(Perdrogen 30% wt/wt solution; Sigma Aldrich, MI, Italy) for 16 h or cultured in DMEM with a concentration of 4500 mg/L of glucose. KCs and FBs of DB pts and HS, and HaCaT cells were treated with 400 UI/ml CAT overnight. This dose was used to resemble the concentration present in topical pharmaceutical preparations to accelerate wound repair [26].

Bone marrow-derived macrophages (BMDM) were polarized to M1 subtype with LPS (10 ng/ml) for 2 h or 8 h and towards M2 subtype with IL-4 (20 ng/ml) for 24 h. BMMP were treated with 1 μM miR-200c inhibitor (LNA-locked nucleic acid anti miR-200c) for 24 h and then stimulated with LPS for the last 2 h.

miRCURY LNA™ microRNA inhibitors of miR-200c or scramble sequence were purchased by Exiqon (Massachusset, USA). Shorter oligonucleotides are internalised much more efficiently via natural cellular uptake mechanisms [30], which explains why shorter LNA™ antisense oligonucleotides are significantly more potent when added directly to cell cultures without transfection reagent (gymnotic delivery).

### Lentiviral infection

Stable expression of miR-200c (in KCs and FBs of HS) and anti-miR-200c (in KCs and FBs of DB), was generated by lentiviral infection. These viruses were produced as previously described [31]. In summary, cells were infected with lentiviral particles for 2 h and then were recovered in complete fresh medium for 24 h. Afterward, infected cells were selected by puromycin-containing medium (Sigma Aldrich, MI, Italy) for 72 h. miR-200c overexpression was controlled by quantitative real-time PCR (RT-qPCR) (see methods below).

### Oxidative stress

Dihydroethidium (DHE) staining is used to measure ROS including both superoxide and peroxide production. ROS were evaluated by incubating the cells or frozen tissues with 2 μM DHE (Sigma-Aldrich, MI, Italy) at 37 °C for 15 min. Then, cells and tissues were fixed with 4% paraformaldehyde and nuclei were stained with DAPI (1:10,000 in PBS; Sigma-Aldrich, MI, Italy). Images were acquired with the ApoTome System (Zeiss, MI, Italy) connected with an Axiovert200 inverted microscope (Zeiss, MI, Italy); image analysis was performed with ZEN software (Zeiss, MI, Italy).

### miRNAscope

miR-200c histologic localization was detected by chromogenic in situ hybridization (ISH) using miRNAscope high-definition (HD) RED procedure (Advanced Cell Diagnostics [ACD], Hayward, CA), according to the manufacturer’s protocol as in [32]. Briefly, OCT tissues were sectioned at 10 μm slices using a cryostat and mounted on Superfrost Plus slides. Successively, sections were dehydrated and post fixated according to the protocol, then peroxidase and protease were applied. Tissue sections were hybridized with the custom designed probe against miR-200c-3p (SR-hsa-miR-200c3p S1 728531-S1). Small RNA integrity and signal specificity were confirmed with a positive control probe U6 (PN 727871-S1), and a negative control probe scramble (PN 727881-S1). Then, the amplification cascade was performed as stated in the manufacturer’s protocol and detected by using Fast Red solution, afterwards sections were counterstained with DAPI. Images were scanned using Axioplan 2 microscope (Carl Zeiss AG, Oberkochen, Germany) and analysed using Zen 3.1 Blue software. For quantification, the analysis of the chromogenic miRNAscope substrate was done using image J software, subtracting miR-scramble positive signal areas and normalizing to U6 snRNA signal. For each tissue section five different areas were quantified and then mediated.

### Immunohistochemistry

Paraffin-embedded sections were obtained from skin biopsies of HS or DB pts. 5 μm sections were dewaxed and rehydrated. After quenching endogenous peroxidase, achieving antigen retrieval, and blocking non-specific binding sites, sections were incubated with the following primary antibodies: anti-ZEB1 (H-102), anti-SIRT1 (H-300), anti-FOXO1 (H-128), anti-SHC (Transduction Laboratories, New Jersey, USA), and anti-phospho p66Shc-pSer36 (6E10) (Abcam, Cambridge, UK). Either rabbit or mouse IgG isotype control (Santa Cruz, Dallas, TX, USA) was used at the same concentration of primary antibodies. Secondary biotinylated polyclonal Abs and staining kits were obtained from Vector Laboratories. Immunoreactivity was visualized with peroxidase reaction using 3-amino-9- ethylcarbazole (AEC, Vector Laboratories, Burlingame, CA) in H_2_O_2_ and specimen counterstained with hematoxylin. As a negative control, primary Abs was omitted. Stained sections were analysed with the AxioCam digital camera coupled to the Axioplan 2 microscope (Carl Zeiss AG, Oberkochen, Germany). Primary antibodies staining intensity were evaluated by quantitative analysis (Image J color deconvolution) in three adjacent fields of each section by two independent observers, blinded to the status of the specimens. The percentage of IHC positivity areas was shown in scatter plots graphs.

### Cytotoxicity assay

KCs and FBs of HS or DB pts were infected with lentiviruses encoding either miR-200c or anti-miR-200c and treated with 400 UI/ml of CAT for 16 h. This dose was used to resemble the concentration present in topical pharmaceutical preparations to accelerate wound repair [26]. CellTox Green Cytotoxicity Assay (Promega, MI, Italy) was used to test cytotoxicity. The fluorescence was measured at 500 nm Ex/530 nm Em at EnSight microplate reader (Perkin Elmer, Massachusetts, USA).

### Apoptosis analysis

Apoptosis of FBs and KCs was evaluated using the FITC Annexin V/propidium iodide (PI) apoptosis detection kit (BD Biosciences, Milan, Italy). Viable, necrotic, and apoptotic cells were analysed by Accuri C6 Flow cytometer (BD) equipped with Cell Quest software. The percentage of Annexin V + , PI + , and Annexin V^−^/PI + cell populations was evaluated in cultures of FBs and KCs of HS infected with lentiviruses encoding either miR-scramble or miR-200c at 16 h.

### Proliferation assay

Proliferation was assayed by CyQuant cell proliferation assay kit (C7026, Invitrogen, Monza, Italy). Detection reagent was added to the wells for 1 h at 37 °C and then fluorescence was measured at 508 nm Ex/527 nm Em with the EnSight microplate reader (Perkin Elmer, Waltham, MA, USA).

### Western blot analysis

Cells were lysed in a buffer containing 100 mM Tris (pH 6.8), 20% glycerol, and 4% sodium dodecyl sulphate (SDS). Protein concentration was determined by BCA protein assay kit (Pierce, Massachusetts, USA). Then, dithiothreitol 200 mM was added and lysates were boiled for 5 min. Proteins were separated by SDS–polyacrylamide gel electrophoresis (SDS–PAGE) and transferred to nitrocellulose membrane by standard procedures. The membranes were blocked with 5% non-fat dry milk powder in 0.05% Tween 20 phosphate-buffered saline (PBS-T) for 1 h. Immunodetection was performed by incubating the membranes with different primary antibodies overnight at 4 °C. After four washes with PBS-T, the membranes were incubated with secondary antibody conjugated with horseradish peroxidase for 1 h. After washes, blots were developed with Amersham-ECL-Plus and exposed to ChemiDoc (Bio-Rad, Hercules, CA, USA). Protein levels were evaluated by densitometric analysis using Image Lab Software (Bio-Rad, Hercules, CA, USA). Protein expression was normalized for α-tubulin and vinculin protein levels. The following primary antibodies were used to detect the proteins of interest: anti-ZEB1 (H-102), anti-SIRT1 (H-300), anti-FOXO1 (H-128), anti-SHC (Transduction Laboratories, New Jersey, USA), anti-vinculin (7F9) (Santa Cruz Biotechnology, Dallas, TX, USA), anti-phosphop66-pSer36 (6E10) (Abcam, Cambridge, UK), anti-α-tubulin (Ab-1) (Oncogene Research Products, La Jolla, CA, USA).

### RNA Extraction and analysis

Total RNA, from skin samples, HaCaT, FBs, and KCs, was extracted using QIAzol (Qiagen s.r.l., MI, Italy). miRNA levels were analysed using the TaqMan quantitative real-time PCR (qRT-PCR) and quantified with the QuantStudio5 real-time PCR (Thermo Fisher Scientific, Massachusetts, United States). Primers for miR-200a, miR-200b, miR-200c, miR-141, miR-429 and the reagents for reverse transcriptase and qPCR reactions were all obtained from Applied Biosystems (Monza, Italy). miRNA expression levels in each sample were normalized to miR-16 and to U6 small RNA expression. Relative expression in the fold was calculated using the comparative Ct method (2–ΔΔCt) [33]. cDNA was generated by the SuperScript First-Strand Synthesis System (Invitrogen, Monza, Italy), and real-time PCR was performed with the SYBR-GREEN RT-qPCR method (Qiagen s.r.l., MI, Italy) using QuantStudio5 Realtime-PCR. Human mRNA expression was normalized to Beta 2 microglobulin, RPL13 and 18S rRNA obtaining the same results. TATA-box binding protein (TBP), was used in BMDM experiments, since this gene is considered one of the best genes for RT-qPCR normalization upon LPS stimulation [34]. Relative expression was calculated using the comparative Ct method (2–ΔΔCt). The following primers were used for RT-qPCR:

human 18S.

Forward 5′CGAGCCGCCTGGATACC-3′

Reverse 5′CATGGCCTCAGTTCCGAAAA-3′

human ZEB1.

Forward: 5′-GGGAGGAGCAGTGAAAGAGA-3′

Reverse: 5′-TTTCTTGCCCTTCCTTTCTG-3′

human SIRT1:

Forward: 5′-AAATGCTGGCCTAATAGAGTGG-3′

Reverse: 5′-TGGCAAAAACAGATACTGATTACC-3′

human FOXO1:

Forward: 5′-AAGGGTGACAGCAACAGCTC-3′

Reverse: 5′-TTCCTTCATTCTGCACACGA-3′

human SHC:

Forward: 5′-GTATGTGCTCACTGGCTTGC-3′

Reverse: 5′-CAAAGCGGTGATCCTTAGTCC-3′

murine TBP.

Forward: 5′-CTGGAAATTGTACCGCAGCTT-3′.

Reverse: 5′-TCCTGTGCACACCATTTTTC-3′.

murine SIRT1:

Forward: 5′-ACAGTGAGAAAATGCTGGCC-3′.

Reverse: 5′-GTATACCTCAGCACCGTGGA-3′.

murine TNF-α.

Forward: 5′-TCTTCTCATTCCTGCTTGTGG-3′.

Reverse: 5′-CACTTGGTGGTTTGCTACGA-3′.

murine IL-6.

Forward: 5′-TCCTCTCTGCAAGAGACTTCC-3′.

Reverse: 5′-TTGTGAAGTAGGGAAGGCCG-3′.

murine IL-1β.

Forward: 5′-GACCTTCCAGGATGAGGACA-3′.

Reverse: 5′-TCCATTGAGGTGGAGAGCTT-3′.

murine i-NOS (NOS2).

Forward: 5′-CCATCATGAACCCCAAGAGT-3′.

Reverse: 5′-CATCCAGAGTGAGCTGGTAGG-3′.

### Scratch assay

The linear WH assay is an in vitro technique used to analyse cell migration and proliferation. By physical exclusion, an area free of cells in a confluent monolayer of cells is created. Exposure to the cell-free area causes the cells themselves to migrate into the gap. To perform this technique, the Culture-Insert 2 Well in μ-Dish 35 mm (Ibidi GmbH, Gräfeling, Germany), consisting of two wells separated by a 500 μm thick wall, as cell support was used. Each well can contain a volume of cells equal to 70 μl. The monitoring of the wound, corresponding to the change over time of the area covered by cells, is carried out through a camera connected to a microscope. The scratch assay was performed in serum free media. From each acquired image, cell migration and proliferation were quantified by measuring the distance between the ends of the cut in 5 randomly selected points using the ImageJ software. The data obtained were normalized respect to the width of the control cells fixed immediately after the mechanical removal of cells from the monolayer.

### Diabetic mice and wound closure analysis

All experimental procedures complied with the Guidelines of the Italian National Institutes of Health and with the Guide for the Care and Use of Laboratory Animals (Institute of Laboratory Animal Resources, National Academy of Sciences, Bethesda, MD) and were approved by the institutional Animal Care and Use Committee. Db/db 3 months old male mice were purchased from Charles River Laboratories Italia Srl (Calco (LC), Italy). A 6-mm-diameter full-thickness excisional wound was performed on the dorsum of diabetic mice previously depilated. At days 0 and every 3 days after injury until wound closure, 50 μl of a mixture of 30% Pluronic F-127 gel containing either 2 nmoles LNA-scramble or anti-miR-200c (0.04 nmoles/μl) in presence or absence of 400 UI of CAT (8UI/μl), was applied to the wound. The CAT concentration is the same used in topical pharmaceutical preparations to accelerate wound repair [26]. Wound areas were photographed at days 0, and every three days thereafter, until closure and measured, using a blinded analysis, by a computer-assisted image analyzer (Image J software). The healing rate expressed as the percentage of initial area.

In vivo miRCURY LNA™ microRNA inhibitors of miR-200c or scramble sequence were purchased by Exiqon (Massachusset, USA). LNA™ antisense oligonucleotides are significantly more potent when added directly when administered to animals in vivo [30].

### Statistical analysis

All data are expressed as means ± standard error (SEM) from at least 3 independent experiments. Because of the novelty of the study, whose primary objectives are mainly descriptive and exploratory, the minimum sample size has not been predetermined. Each variable was checked for normality distribution by the D’Agostino and Pearson omnibus normality test. The difference between the two groups was compared either by the two-tailed Mann–Whitney or Wilcoxon rank sum test for non-parametric groups or by two-tailed Student’s t test for parametric variables using GraphPad Prism software (Version 5.0). Differences among three or more groups were tested by one-way Anova and Bonferroni post-hoc test. The mice WH assay was modelled with a logistic nonlinear mixed-effects model and fit using the R (version 4.2.0) nlmer function from the lme4 package [35].

## Results

### miR-200c is upregulated by ROS in HaCaT and DFU skin biopsies

We previously showed that oxidative stress upregulates miR-200c in different cell types, thus we tested whether different ROS stimuli i.e. hydrogen peroxide (H_2_O_2_), ultraviolet radiations (UV), and high glucose (HG), modulate miR-200c in immortalized keratinocytes (HaCaT).

**Fig. 1:**
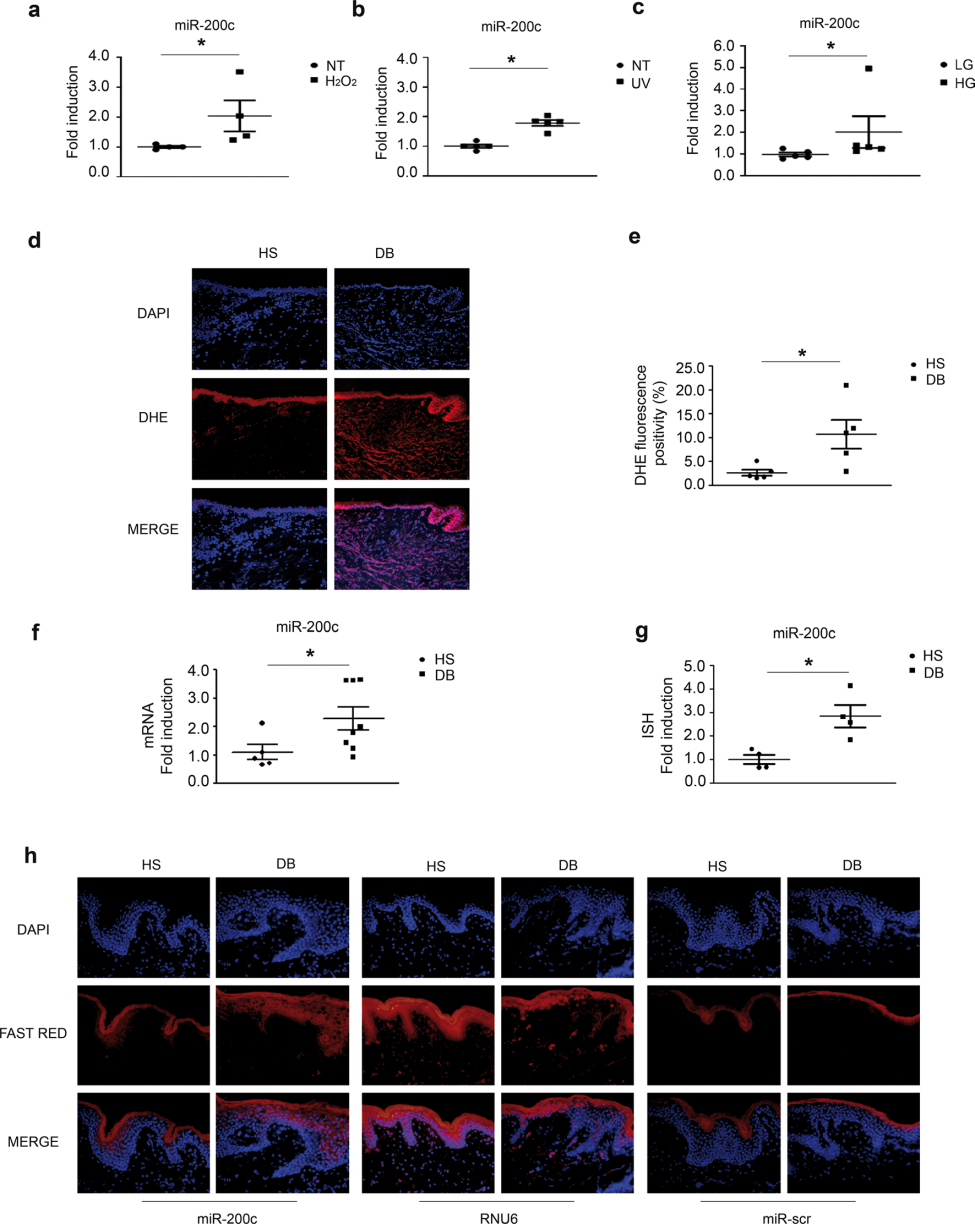
**miR-200c is upregulated by oxidative stress in HaCaT and skin biopsies of pts with DFU.** **a** HaCaT cells were exposed to 400 μM H_2_O_2_ for 16 h. Then total RNA was extracted and assayed for miR-200c expression levels by RT-qPCR. (N = 4; *p < 0.05). **b** HaCaT were treated or not (w/o UV) with 50 J/m^2^ of UV light. After 16 h in serum-free media, total RNA was extracted and assayed for miR-200c expression levels by RT-qPCR (N = 5; * p < 0.05). **c** HaCaT cells were grown in low glucose DMEM (containing 5 mM glucose) supplemented with 25 mM high glucose medium for 3 days, 25 mM mannitol was used as isosmotic control. Then miR-200c expression was evaluated (N = 5; *p < 0.05). **d** Skin Biopsies derived from pts with DFU (DB) undergoing major amputations and from normoglycemic HS pts undergoing saphenectomies, used as controls, were assayed for oxidative stress by DHE fluorescence. Representative images showed that skin tissues of DFU pts display higher oxidative stress. **e** Scatter plot representing the percentage of DHE fluorescence positivity (N = 5 HS, N = 6 DB; *p < 0.05). **f** Total RNA extracted from biopsies of DB pts and HS controls was assayed for miR-200c levels (N = 5 HS; N = 8 DB; *p < 0.05). **g** Scatter plot representing ISH quantification of five different areas per tissue sample, subtracted of miR-scramble signal and normalized *vs* U6 ISH fluorescence positivity. Data are presented as fold induction *vs* ctrl pts (N = 4; *p < 0.05). **h** Representative in situ staining of miR-200c (Red) (left panels) in skin tissues of DB undergoing major amputations and from HS normoglycemic pts controls. In situ staining of U6 snRNA (Red) was used as normalization control (central panels). miR-scramble (Red) was used as negative control (right panels). Skin tissues were counterstained with DAPI (blue). miR-200c in situ staining was higher in DB skin compared to HS one

We found that HaCaT treatment with 400 μM H_2_O_2_ for 16 h induced miR-200c expression levels of approximately two-fold (Fig. 1a). Moreover, miR-200c expression levels were analysed after 16 h of treatment with 50 J/m^2^ UV light, and in this case, miR-200c was similarly induced (⁓twofold) (Fig. 1b). Finally, we treated HaCaT cells with high glucose concentration (30 mM) for 3 days, and an increase of miR-200c was observed (Fig. 1c). DM is known to increase ROS in a variety of tissues, including the skin [36]. We confirmed this data in skin biopsies of pts undergoing major lower extremity amputation *vs* skin biopsies of HS undergoing saphenectomy. DFU skin samples exhibited more ROS than HS ones, as evaluated by red/ox-sensitive fluorescent probe DHE staining (Fig. 1d, e). miR-200c expression levels in skin biopsies of DFU pts was up-regulated *vs* HS (Fig. 1f); whereas the other miR-200 family members were not modulated (Additional file1: Fig. S1a). Notably, in situ hybridization (ISH) confirmed higher miR-200c expression in DFU *vs* HS skin biopsies (Fig. 1g, h) and the induction achieved in DFU samples was comparable to the one found at the RNA level (⁓threefold). ISH showed that miR-200c was highly expressed in the epidermis compared to dermis layer, and the cellular localization was predominantly perinuclear and cytoplasmatic (Fig. 1h, left panels). The ISH was normalized to RNU6 expression levels (Fig. 1h, central panels) and subtracted of miR-scramble signal, used as negative control (Fig. 1h, left panels).

### miR-200c is up-regulated in inflammatory M1 macrophages and its inhibition decreases the expression of M1 genes

**Fig. 2:**
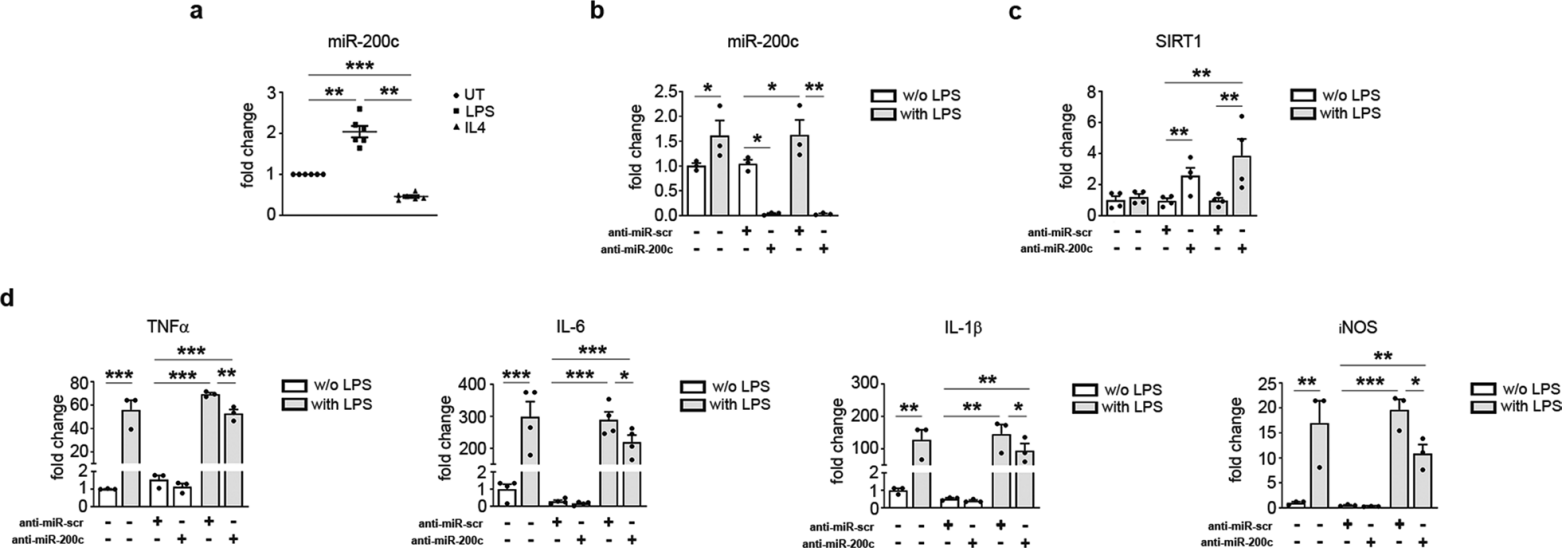
**miR-200c is upregulated in macrophages upon M1 polarization and its inhibition decreases M1 genes.** **a** BMDM were polarized towards M1 and M2 phenotype by LPS (10 ng/ml) and IL-4 (20 ng/ml), respectively, and compared to untreated (UT) macrophages. Total RNA was extracted and miR-200c levels were measured by RT-qPCR. MiR-200c was increased in M1 and decreased in M2 macrophages (N = 6; **p < 0.01; ***p < 0.001). **b, c, d** BMDM were treated with LNA anti miR-scramble (scr) or anti-miR-200c (1 μM) for 24 h, stimulated with LPS (10 ng/ml) for 2 h and compared to unstimulated macrophages. Then total RNA was extracted. **b** miR-200c levels were measured by RT-qPCR in total RNA extracted from BMDM. miR-200c was strongly decreased by LNA anti-miR-200c treatment both under basal and LPS treatment (N = 3; *p < 0.05; **p < 0.01). **c** RT-qPCR of a miR-200c target gene, SIRT1, that was increased in BMDM anti-miR-200c treated cells, both under basal and LPS treatment (N = 4; *p < 0.05; **p < 0.01). **d** RT-qPCR of M1-inflammatory genes: TNFα L-6, IL-1β, **i**NOS. All M1 genes were induced by LPS treatment and were decreased by anti-miR-200c in LPS treated BMDM (N = 3–4; *p < 0.05; **p < 0.01; ***p < 0,001)

In chronic ulcers there is a dysregulation in the transition of macrophages from the pro-inflammatory M1 to the anti-inflammatory M2 phenotype, this phenomenon causes the persistence of an active inflammatory condition that contributes to wound closure failure, and it is at the basis of chronicity of the wound itself [37]. In order to understand whether miR-200c is differently expressed in M1 *vs* M2 macrophages, we analysed miR-200c levels in BMDM induced to polarize towards M1 macrophages with LPS treatment, and to M2 with IL-4 treatment. Interestingly, we found an increase of miR-200c in M1 macrophages and a decrease in M2 (Fig. 2a). Then, we treated BMDM with anti-miR-200c LNA oligos, given in the media without any transfection reagent, and afterwards we induced M1 polarization with LPS. We observed in M1 macrophages a decrease of miR-200c levels (Fig. 2b) and an increase of SIRT1, a miR-200c target, indicating the efficacy of internalization (Fig. 2c). Moreover, we observed a decrease of M1-inflammatory genes, i.e. IL-6, IL-1β, TNFα and iNOS (Fig. 2d), demonstrating a beneficial role of miR-200c inhibition in decreasing inflammatory pathways and immune cell reprogramming.

### miR-200c targets are downregulated and p66Shc Ser-36 phosphorylation is increased in skin biopsies, FBs and KCs of DFU pts

**Fig. 3:**
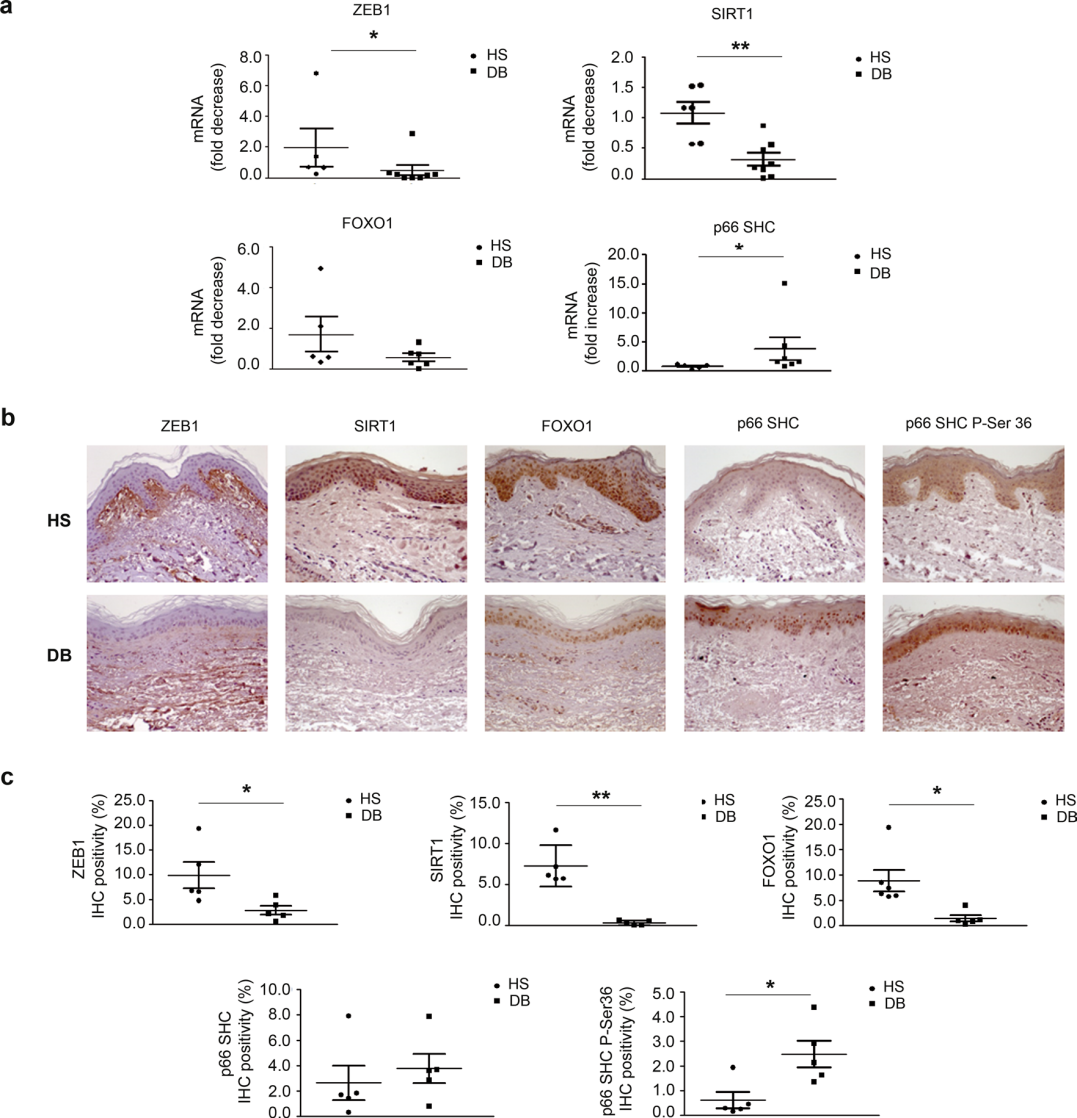
**miR-200c targets are downregulated and p66 Shc Ser-36 phosphorylation is increased in human skin biopsies of DFU pts.** **a** Total RNA extracted from biopsies of DB and normoglycemic controls (HS). ZEB1, SIRT1, FOXO1 and p66Shc mRNA were quantified by RT-qPCR (N = 5–6 HS, N = 6–8 DB *p < 0.05; **p < 0.01). **b** Representative immunohistochemistry (IHC) of ZEB1, SIRT1, FOXO1, P66Shc-P-Ser36 and P66Shc. IHC levels were evaluated in paraffin-embedded biopsies of skin isolated by patient with DFU compared to HS. **c** Scatter plots representing the IHC quantification of ZEB1, SIRT1, FOXO1, P66Shc-P-Ser36 and P66Shc of DFU compared to HS. IHC was quantified by Image J colour deconvolution of three adjacent fields for each section. Graphs showed IHC positivity area, expressed in percentage of positivity (N = 5 HS, N = 5–6 DB; *p < 0.05)

**Fig. 4:**
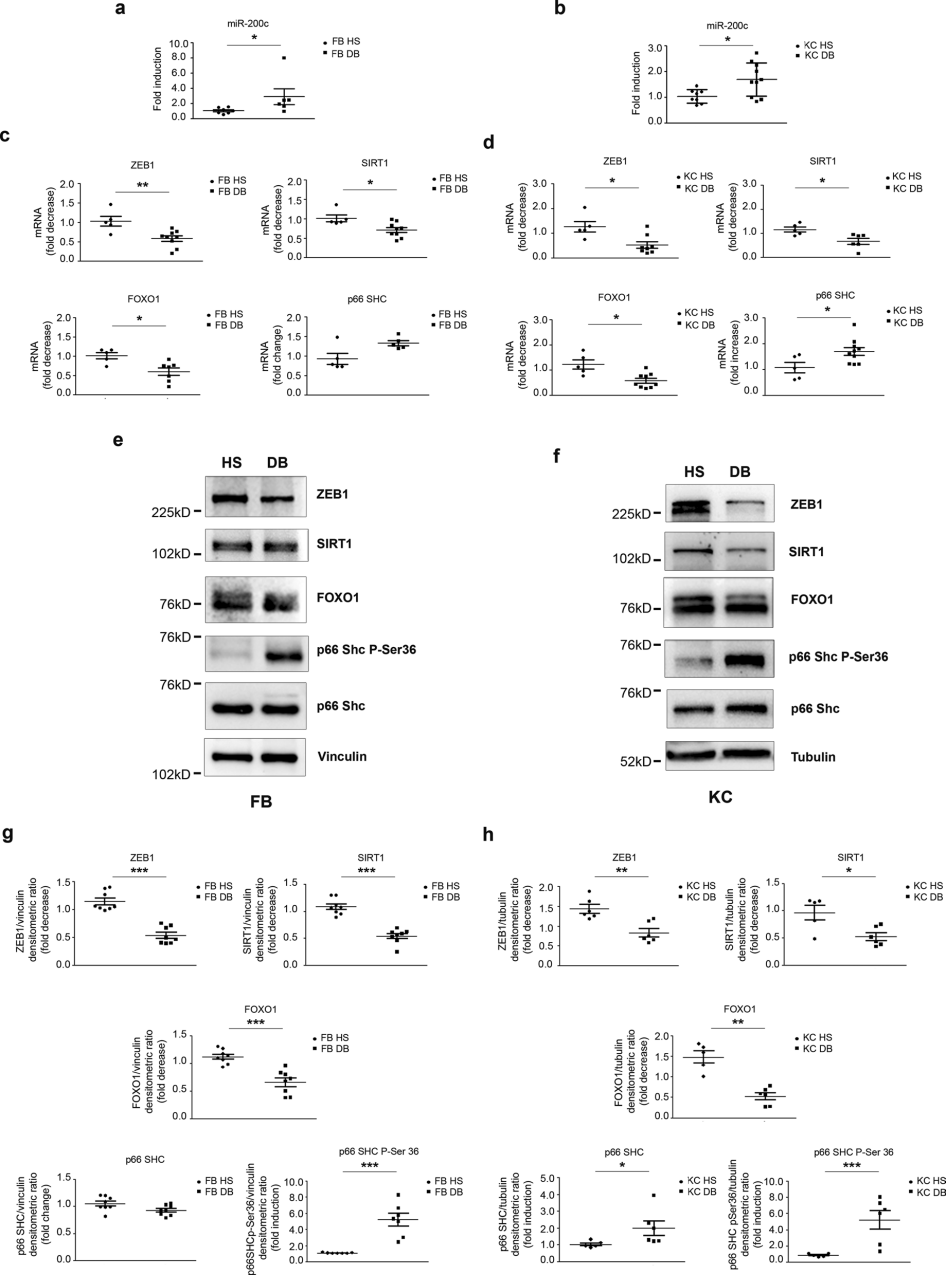
**miR-200c is upregulated in DFU FBs and KCs and its targets are downregulated while p66Shc Ser-36 phosphorylation is decreased.** Total RNA was extracted from FBs and KCs isolated from biopsies of DFU or biopsies of normoglycemic pts (HS). **a** miR-200c expression levels were induced in FBs isolated by biopsies of pts with DFU (FB DB) compared to HS (N = 7 FB HS, N = 8 FB DB; *P < 0.05). **b** miR-200c expression levels were induced in KCs isolated by biopsies of pts with DFU (KC DB) compared to HS (N = 8 KC HS, N = 10 KC DB; *P < 0.05). **c** ZEB1, SIRT1 and FOXO1 mRNA expression levels were downmodulated in FBs extracted by skin biopsies of pts with DFU *vs* healthy control subjects, while P66Shc mRNA was induced, albeit not significantly (N = 5 FB HS, N = 7–9 FB DB *p < 0.05; **p < 0.01). **e** ZEB1, SIRT1 and FOXO1mRNA expression levels were decreased in KCs isolated by skin biopsies of pts with DFU *vs* healthy control subjects, while P66Shc mRNA was induced (N = 5 KC HS, N = 7–8 KC DB *p < 0.05). **e–f** Representative western blots of ZEB1, SIRT1, FOXO1, P66Shc-P-Ser36 and P66Shc of DFU compared to HS ones of FBs and KCs, respectively. **g–h** Expression levels of ZEB1, SIRT1, FOXO1, P66Shc-P-Ser36 and P66Shc proteins were evaluated by densitometric analysis and normalized by either vinculin or α-tubulin protein levels, as indicated (N = 8 FB HS, N = 8 FB DB; N = 5–6 KC HS, N = 6 KC DB; *p < 0.05; **p < 0.01; ***p < 0.001). Source data are available for this figure: Additional file1: Fig.S6

In light of the increased miR-200c expression in DFU samples, we assayed relevant miR-200c direct targets in biopsies. ZEB1, SIRT1 and FOXO1 mRNA were downregulated, but the latter did not reach statistical significance (Fig. 3a); whereas p66Shc mRNA was upregulated in agreement with ROS increase (Fig. 3a). Immunohistochemistry (IHC) analyses revealed that ZEB1, SIRT1, FOXO1 proteins were lower in DFU (Fig. 3b, c). Moreover, p66Shc was increased in DFU *vs* HS skin biopsies, but it did not reach statistical significance, whereas p66Shc P-Ser36 was increased (Fig. 3b, c). miR-200c was also upregulated in both FBs and KCs extracted from DFU biopsies (Fig. 4a, b). The other miR-200 family members were slightly increased in FBs (Additional file1: Fig.S1b) and KCs, but only miR-200a and miR-141 reached statistical significance in KCs (Additional file1: Fig.S1c). For this reason, we focused on miR-200c role in chronic wounds, as it was consistently upregulated in both FBs and KCs. ZEB1, FOXO1 and SIRT1 mRNA expression levels were downregulated in both FBs and KCs of DFU pts (Fig. 4c, d); whereas p66Shc mRNA levels in FBs and KCs of DFU pts were higher than in HS, statistical significance was reached in KCs (Fig. 4c, d). Moreover, ZEB1, SIRT1 and FOXO1 proteins were lower in FBs (Fig. 4e, g) and KCs (Fig. 4f, h) of DFUs pts compared to HS. We confirmed that p66Shc P-Ser36 was upregulated in both FBs and KCs, and a slight significant increase in p66Shc was also found in KCs but not in FBs (Fig. 4e–h) (Uncropped images of Western blots are shown in Additional file1: Fig.S6).

### CAT inhibits ROS-induced miR-200c upregulation

**Fig. 5:**
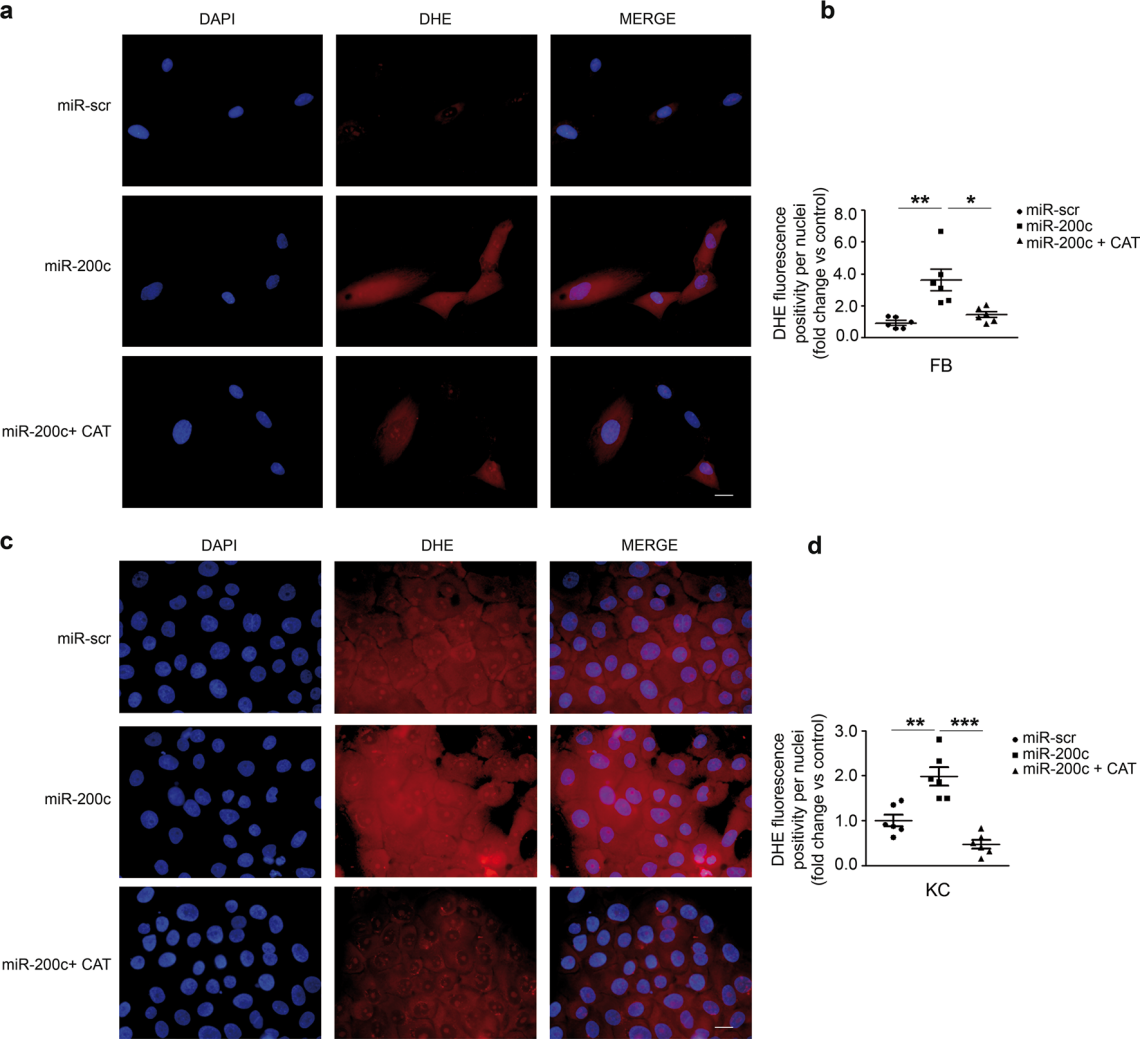
**CAT treatment decreased miR-200c-induced oxidative stress.** Primary human skin FBs from HS were infected either with a lentivirus encoding miR-200c or with a control virus. Then cells were treated with 400 UI/ml equine CAT for 16 h. **a** Representative DHE fluorescence of FBs overexpressing miR-200c with or without CAT. Nuclei were stained with DAPI. Scale bar: 10 μm. **b** Scatter plot representing DHE fluorescence positivity per nuclei and expressed as fold change *vs* ctrl (N = 6; *p < 0.05; **p < 0.01). **c** Primary human skin KCs were infected either with a lentivirus encoding miR-200c or with a control virus. Then cells were treated with 400 UI/ml equine CAT for 16 h. Representative DHE fluorescence of KCs overexpressing miR-200c with or without CAT. Nuclei were stained with DAPI. Scale bar: 10 μm. **d** Scatter plot representing DHE fluorescence positivity per nuclei and expressed as fold change *vs* ctrl (N = 6; **p < 0.01; ***p < 0.001)

**Fig. 6:**
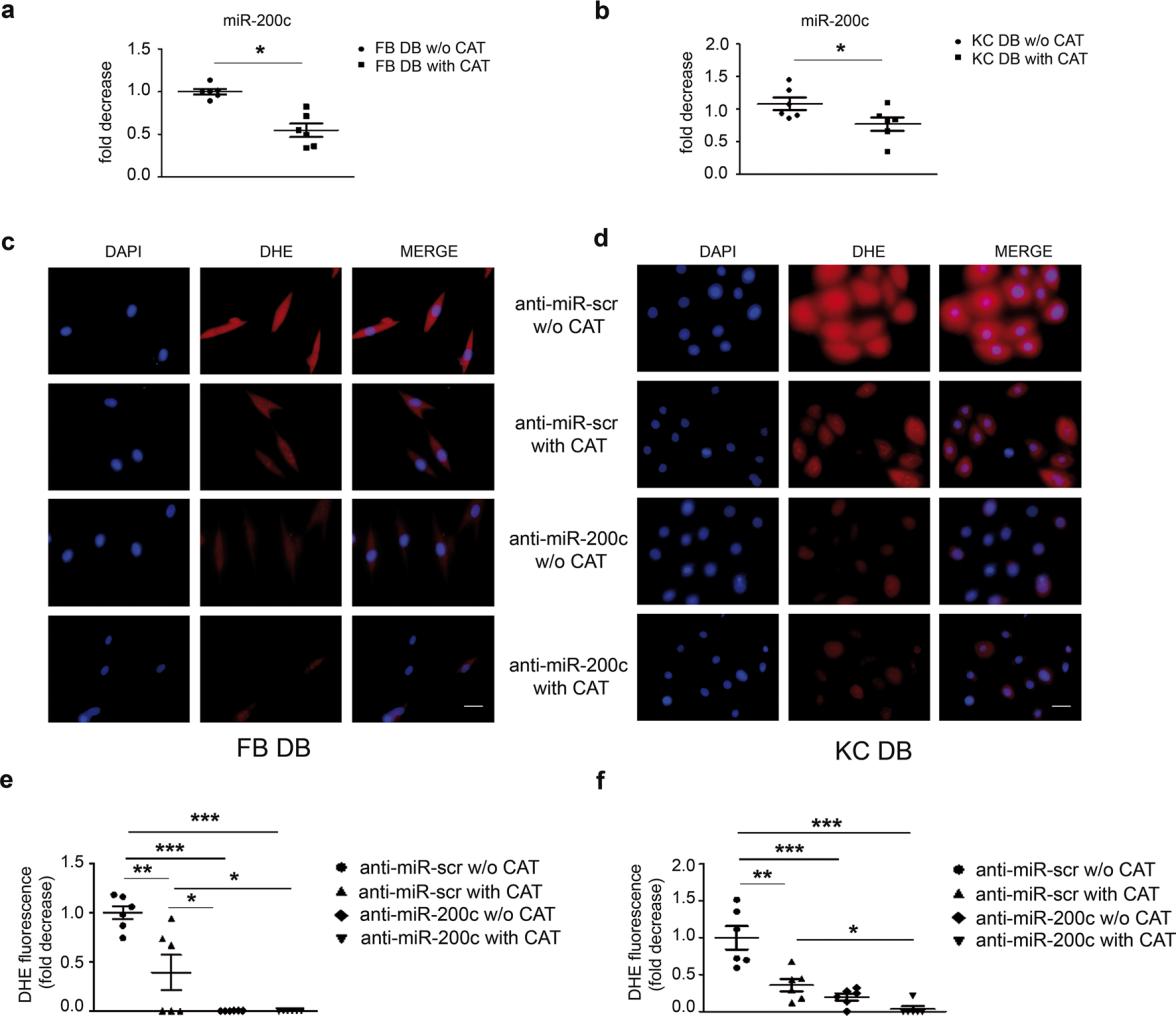
**miR-200c inhibition and CAT treatment downmodulated oxidative stress in FBs and KCs of DFU pts**. **a, b** FBs and KCs of DFU pts were treated with 400 UI/ml for 16 h, then cells were harvested and miR-200c expression levels were quantified by RT-qPCR (N = 6 FB DB, N = 6 KC DB; *p < 0.05). **c**, **d** FBs and KCs of DFU pts were infected either with a lentivirus encoding anti-miR-200c or with a control virus (anti-miR-scr). Then cells were incubated with 400 UI/ml equine CAT for additional 16 h. Representative image of FBs and KCs diabetic pts showed that CAT treatment and anti-miR-200c individually decreased DHE signal compared to anti-miR-scr untreated cells, the co-treatment reduced oxidative stress especially in KC of DFU. **e**, **f** Oxidative stress was assayed by DHE fluorescence which was normalized for the number of DAPI-positive nuclei and expressed as fold decrease *vs* anti-miR-scr untreated cells. Scale bar: 10 μm (N = 6 FB DB; N = 6 KC DB; *p < 0.05; **p < 0.01; ***p < 0.001)

We then examined whether miR-200c induces ROS in FBs and KCs, as previously observed in ECs [5]. Primary FBs and KCs of HS skin biopsies were transduced either with a lentivirus encoding miR-200c or with a control virus (miR-scramble). First, we assayed miR-200c upregulation upon infection (Additional file1: Fig. S2a, b). miR-200c increased in both cell types, but the overexpression was more efficient in FBs, since miR-200c basal expression levels were lower in FBs than in KCs (Additional file1: Fig. S2a, b). We found that miR-200c increased ROS, assayed by DHE, and that the co-treatment with CAT markedly diminished it, in both FBs (Fig. 5a, b) and KCs (Fig. 5c, d). Interestingly, we found that CAT decreased miR-200c in both cells of DFU pts, demonstrating that miR-200c increase is partly due to oxidative stress (Fig. 6a, b). Notably, CAT treatment did not modulate miR-200a/−200b/−141/−429 in both DFU FBs and KCs (Additional file1: Fig.S3a, b). We then examined whether anti-miR-200c reduced ROS in DFU cells and whether co-treatment with CAT could amplify this reduction. To this aim both FBs and KCs of DFU pts were transduced with anti-miR-200c either alone or with CAT. The efficacy of anti-miR-200c treatment on miR-200c expression was confirmed in both DFU FBs and KCs (Additional file1: Fig. S2c, d). Anti-miR-200c reduced ROS better than CAT in FBs (Fig. 6c, e), thus the effect of CAT was not additive in decreasing ROS (Fig. 6c, e). Anti-miR-200c and CAT were similarly effective in decreasing ROS in KCs, and co-treatment was higher than CAT treatment alone (Fig. 6d, f).

These results suggest that anti-miR-200c and CAT co-treatment could be used to reduce ROS in DB skin lesion.

### Anti-miR-200c and CAT upregulate miR-200c targets and decrease p66Shc Ser-36 phosphorylation

**Fig. 7:**
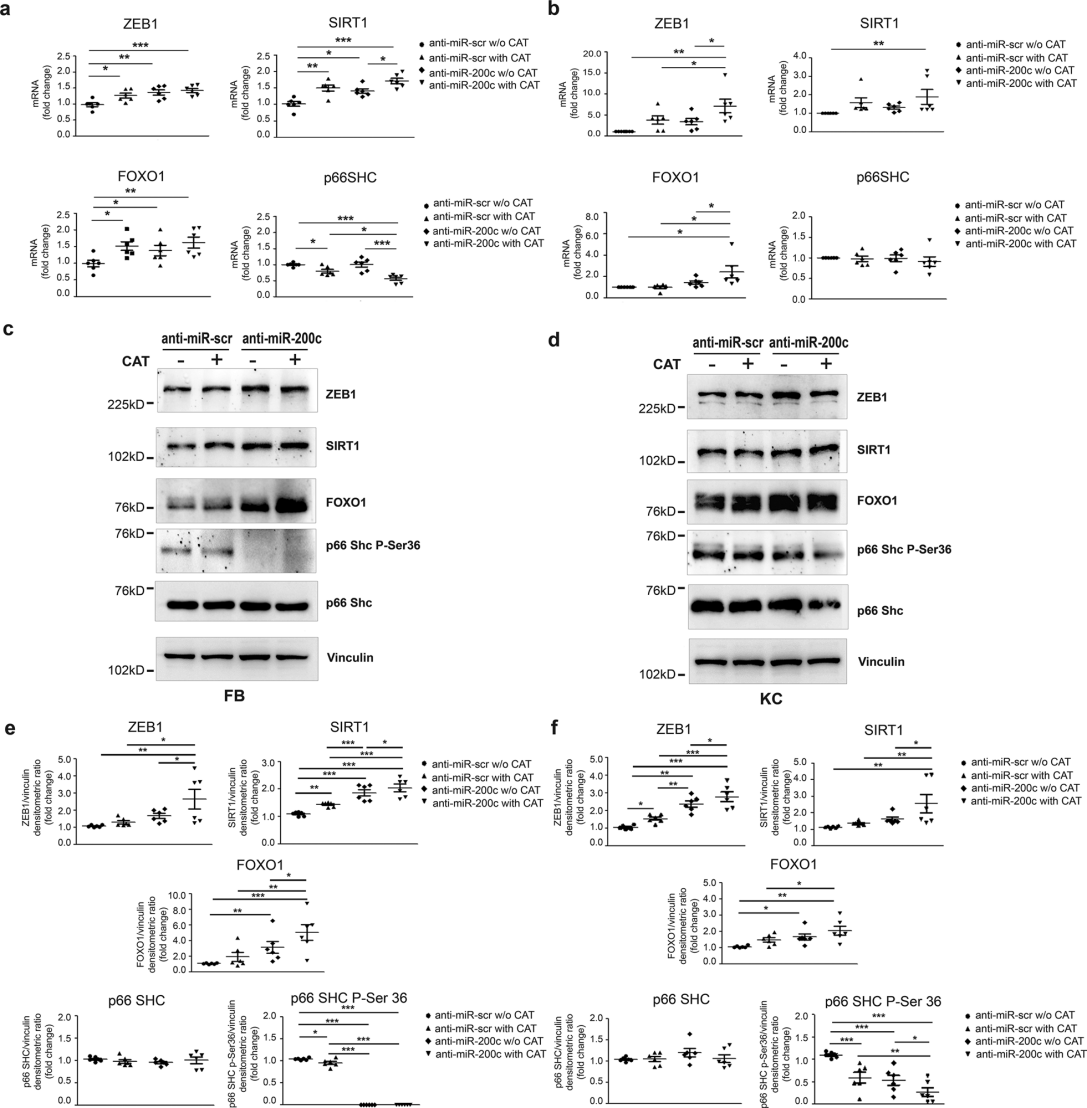
**miR-200c targets are upregulated and p66shc Ser-36 phosphorylation is decreased by anti-miR-200c and CAT treatment in DFU FBs and KCs**. FBs and KCs of DFU pts were infected either with a lentivirus encoding anti-miR-200c or with a control virus. Afterwards cells were incubated with 400 UI/ml CAT for additional 16 h. **a, b** ZEB1, SIRT1, FOXO1 and P66Shc mRNA were quantified by RT-qPCR in FBs and KCs respectively (N = 6; *p < 0.05; **p < 0.01; ***p < 0.001). **c, d** Representative western blots of ZEB1, SIRT1, FOXO1, P66Shc-P-Ser36 and P66Shc in FBs and KCs of DFU pts, respectively. **e, f** Scatter plots showing the expression levels of ZEB1, SIRT1, FOXO1, P66Shc-P-Ser36 and P66Shc proteins were evaluated by densitometric analysis normalized to vinculin protein levels in FBs and KCs (N = 6; *p < 0.05; **p < 0.01; ***p < 0.001). Source data are available for this figure: Additional file1: Fig.S6

Successively, we analysed the effect of anti-miR-200c and CAT on miR-200c molecular targets in both FBs and KCs of DFU pts. Single treatments caused an upregulation of ZEB1, SIRT1, FOXO1 mRNA and a downmodulation of p66Shc mRNA only with CAT treatment in FBs (Fig. 7a). The co-treatment induced a further increase of SIRT1 mRNA vs single treatments and a further decrease of p66Shc mRNA (Fig. 7a). In KCs, single treatments caused a slight, but not significant upregulation of ZEB1, SIRT1 and FOXO1 mRNA, whereas p66Shc mRNA was not modulated (Fig. 7b). The co-treatment induced a significant increase of ZEB1, SIRT1 and FOXO1 mRNA *vs* control, and for ZEB1 and FOXO1 mRNA, also compared to single treatments (Fig. 7b). We then analysed miR-200c targets at protein level. SIRT1 protein expression was increased by CAT treatment *vs* control, anti-miR-200c treatment further increased SIRT1 compared to CAT, and the co-treatment was more efficacious than single treatments in DFU FBs (Fig. 7c, e). A similar trend was observed for ZEB1 and FOXO1 proteins, but reached statistical significance only with anti-miR-200c treatment for FOXO1. The co-treatment increased both ZEB1 and FOXO1 proteins compared to control and *vs* single treatments (Fig. 7c, e). The co-treatment decreased also p66Shc P-Ser36, which was slightly decreased by CAT, and strongly decreased by anti-miR-200c, it was not additive compared to anti-miR-200c, but it was statistically significant *vs* CAT (Fig. 7c, e). In KCs, CAT treatment increased ZEB1 protein *vs* control, anti-miR-200c treatment further increased ZEB1 compared to CAT and the co-treatment was more efficacious than single treatments. A similar trend was visible for SIRT1, but only the co-treatment reached statistical significance *vs* control and single treatments (Fig. 7c, e). FOXO1 protein was increased by anti-miR-200c and co-treatment, and the latter was more effective than CAT on FOXO1 upregulation (Fig. 7c, e). p66Shc P-Ser36 was decreased by single treatments and the co-treatment was more efficacious in decreasing the phosphorylation compared to either treatment alone (Fig. 7c–f). Neither anti-miR-200c, nor CAT, nor their combination influenced p66Shc protein levels in either cell type (Fig. 7c–f) (Uncropped images of Western blots are shown in Additional file1: Fig.S6).

### Anti-miR-200c and CAT decrease cytotoxicity in FBs and KCs of DFU pts

**Fig. 8:**
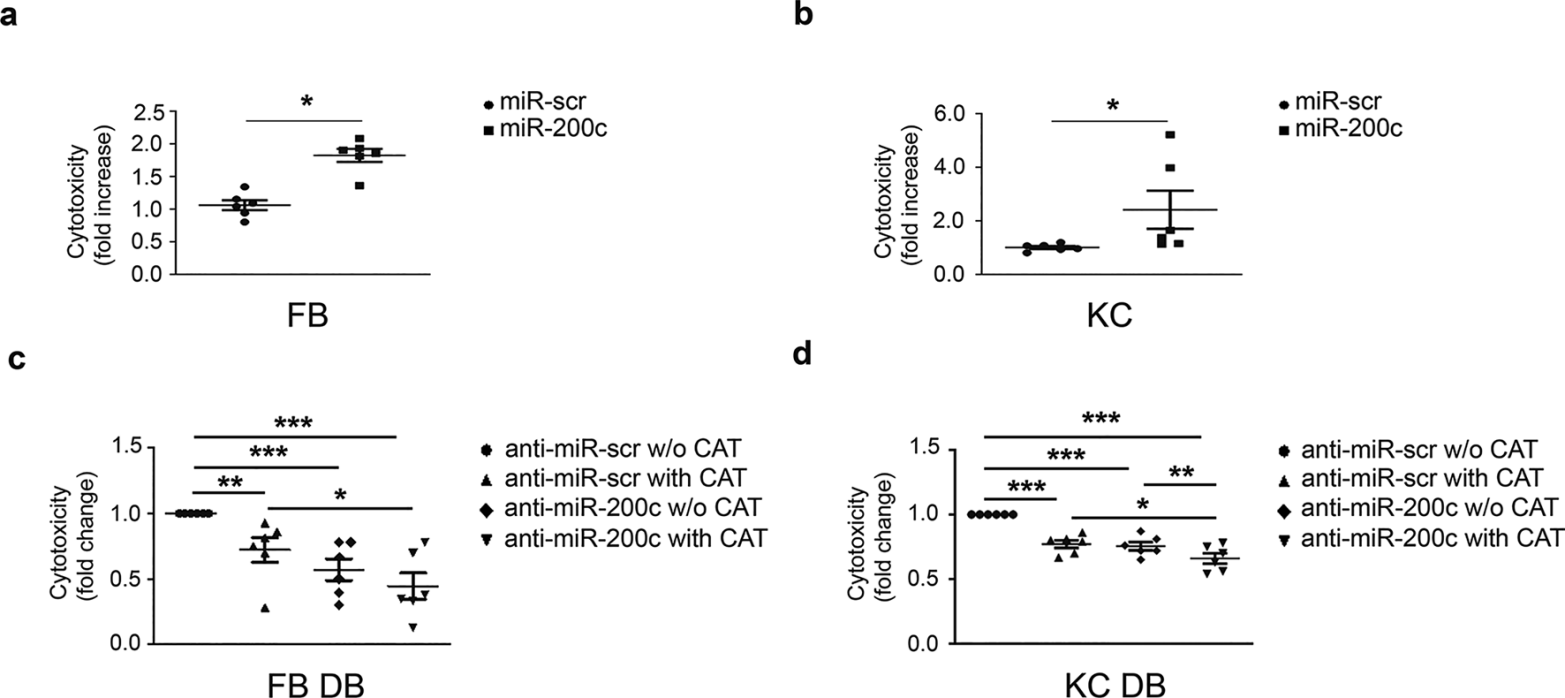
**Anti-miR-200c and CAT treatment decrease DFU FBs and KCs cytotoxicity and their action is synergic.** **a, b** FBs and KCs of HS were infected either with a lentivirus encoding miR-200c or with a control virus. After 16 h cytotoxicity assay was performed. miR-200c overexpression increased cytotoxicity (N = 6; *P < 0.05). **c, d** FBs and KCs of DFU pts were infected either with a lentivirus encoding anti-miR-200c or with a control virus (anti-miR-scr). Afterwards, cells were incubated with 400 UI/ml CAT for additional 16 h. Cytotoxicity assay was performed (N = 6; *p < 0.05; **p < 0.01; ***p < 0.001)

To comprehend the physiological relevance of miR-200c upregulation, FBs and KCs derived from HS were transduced with miR-200c. We found that miR-200c overexpression significantly enhanced cytotoxicity in both FBs and KCs (Fig. 8a, b). To exclude that ROS measurement and gene expression might be indirectly influenced by cell death in miR-200c overexpressing cells, we analysed the percentage of early apoptosis, late apoptosis, necrosis, and survival in both FBs and KCs in detail using Annexin V/PI assay (Additional file 1: Fig. S4a, b, c). An increase in necrosis (from 4.04% to 8.18%), early apoptosis (from 0.42% to 5.97%), late apoptosis (from 0.65% to 4.99%), and a decrease in viable cells (from 94.89% to 80.9%) was observed in FBs upon miR-200c overexpression. In miR-200c overexpressing KCs, we confirmed an increase in the percentage of necrotic cells (from 6.25% to 7.26%), early apoptosis (from 1.74% to 3.95%), and late apoptosis (from 9.02% to 9.54%). The percentage of viable cells decreased from 82.99% to 79.25%. We then evaluated whether single treatments and co-treatments modulated cytotoxicity in DB cells, and we found that CAT and anti-miR-200c alone decreased cytotoxicity and that co-treatment further decreased it in both FBs and KCs of DFU pts (Fig. 8c, d). We also analysed the effect of miR-200c on proliferation. We found that in both FBs and KCs the overexpression of miR-200c by lentiviral transduction decreased proliferation at 48 h (Additional file 1: Fig. S5a, b), in contrast, we failed to see a significant effect on proliferation in FBs and KCs of DFU upon anti-miR-200c and CAT treatments alone and in conjunction (Additional file 1: Fig. S5c, d).

### Anti-miR-200c and CAT accelerate WH in vitro

**Fig. 9:**
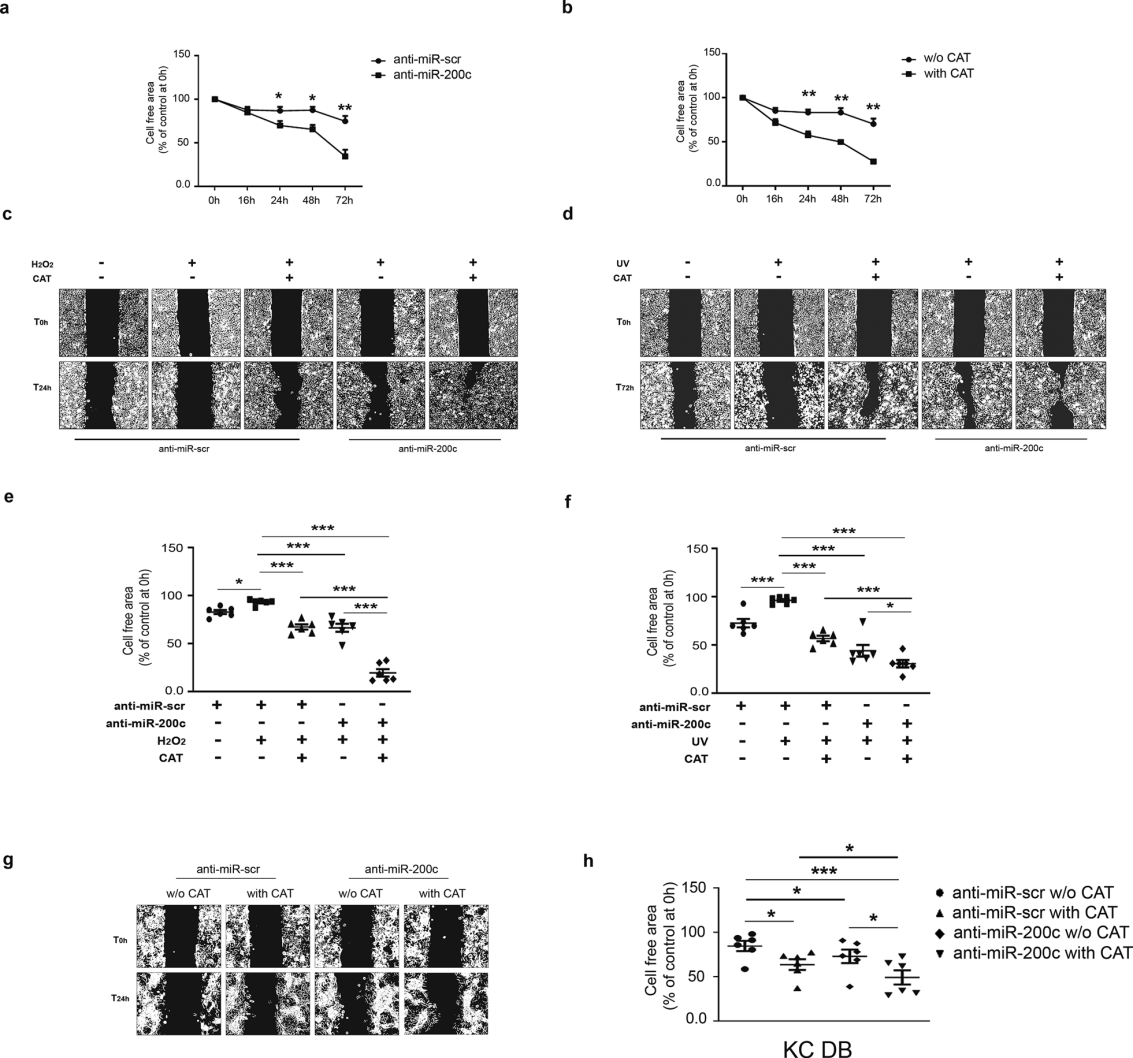
**Anti-miR-200c and CAT accelerate WH upon ROS and in DFU KCs and co-treatment is synergic.** **a** HaCaT cells were infected with either anti-miR-200c or with a control virus (anti-miR-scr) and a scratch assay was performed. Graph showing the % of cell free area *vs* time zero of anti-miR-200c treated cells compared with miR-scramble control at the time points indicated in figure (N = 6; *p < 0.05, **p < 0.01). **b** HaCaT cells were treated with 400 UI/ml CAT for the indicated times during the scratch assay. Graph showing the % of cell free area *vs* time zero of CAT treated cells compared to untreated control at the timepoints indicated in figure (N = 5; **p < 0.01). **c** HaCaT cells were infected with anti-miR-200c or with a control virus. A scratch assay was performed and then cells were treated or not for 16 h with 400 μM H_2_O_2_, afterwards cells were treated or not with CAT for 8 h. Representative image of scratch assay showing the migration process at 24 h. **d** HaCaT cells were infected with either anti-miR-200c or with a control virus, then cells were irradiated with 50 J/m^2^ of UV light. Afterwards, a scratch assay was performed, and the cells were treated or not with 400 UI/ml CAT. Representative images of the scratch assay monitoring cellular migration at 72 h. **e** Scatter plot showing the % of cell free area *vs* time zero at 24 h (N = 6; *p < 0.05; **p < 0.01; ***p < 0.001). **f** Scatter plot showing the % of cell free area *vs* time zero at 72 h (N = 6; *p < 0.05; **p < 0.01; ***p < 0.001). **g** KCs of DFU pts were infected either with a lentivirus encoding anti-miR-200c or with a control virus (anti-miR-scr). Afterwards, cells were incubated with 400 UI/ml equine CAT for additional 24 h. Representative image of a scratch assay that monitored the migration process at 24 h. **h** Scatter plot showing % of cell free area *vs* time zero (N = 6; *p < 0.05; ***p < 0.001)

miR-200c modulates ROS, which play an important role in WH [38–40]. Moreover, different miR-200c targets examined e.g. SIRT1 and p66Shc, affect mechanisms involved in wound closure and in DB WH [41, 42]. Therefore, we examined the role of miR-200c in a WH assay in vitro. We first confirmed that anti-miR-200c downregulates miR-200c expression in HaCaT cells (Additional file1: Fig.S2e). Then we performed a scratch assay in HaCaT transduced with anti-miR-200c or a control sequence. Anti-miR-200c and CAT accelerated WH at all time points starting from 24 h untill the end of the assay at 72 h (Fig. 9a, b). Additionally, we found that anti-miR-200c and CAT accelerated WH in HaCaT either under H_2_O_2_ (Fig. 9c) or UV (Fig. 9d) treatment. Moreover, the co-treatment further ameliorated healing upon both H_2_O_2_ treatment and UV radiation (Fig. 9c–f). We then examined whether anti-miR-200c and CAT modulated WH process of KCs of DFU pts. For this purpose, DFU KCs were infected with lentiviruses expressing either anti-miR-200c or a control sequence and then treated with CAT for 24 h. CAT and anti-miR-200c treatments had a similar effect on WH compared to control cells and co-treatment further improved healing compared to single treatments (Fig. 9g, h). At 24 h we did not observe any change in the proliferation assay (Additional file 1: Fig. S5d), thus the effect on wound healing is most probably due to an increase in migration rather than proliferation.

### Anti-miR-200c and CAT accelerate WH in vivo, in diabetic mice and co-treatment is more effective

**Fig. 10:**
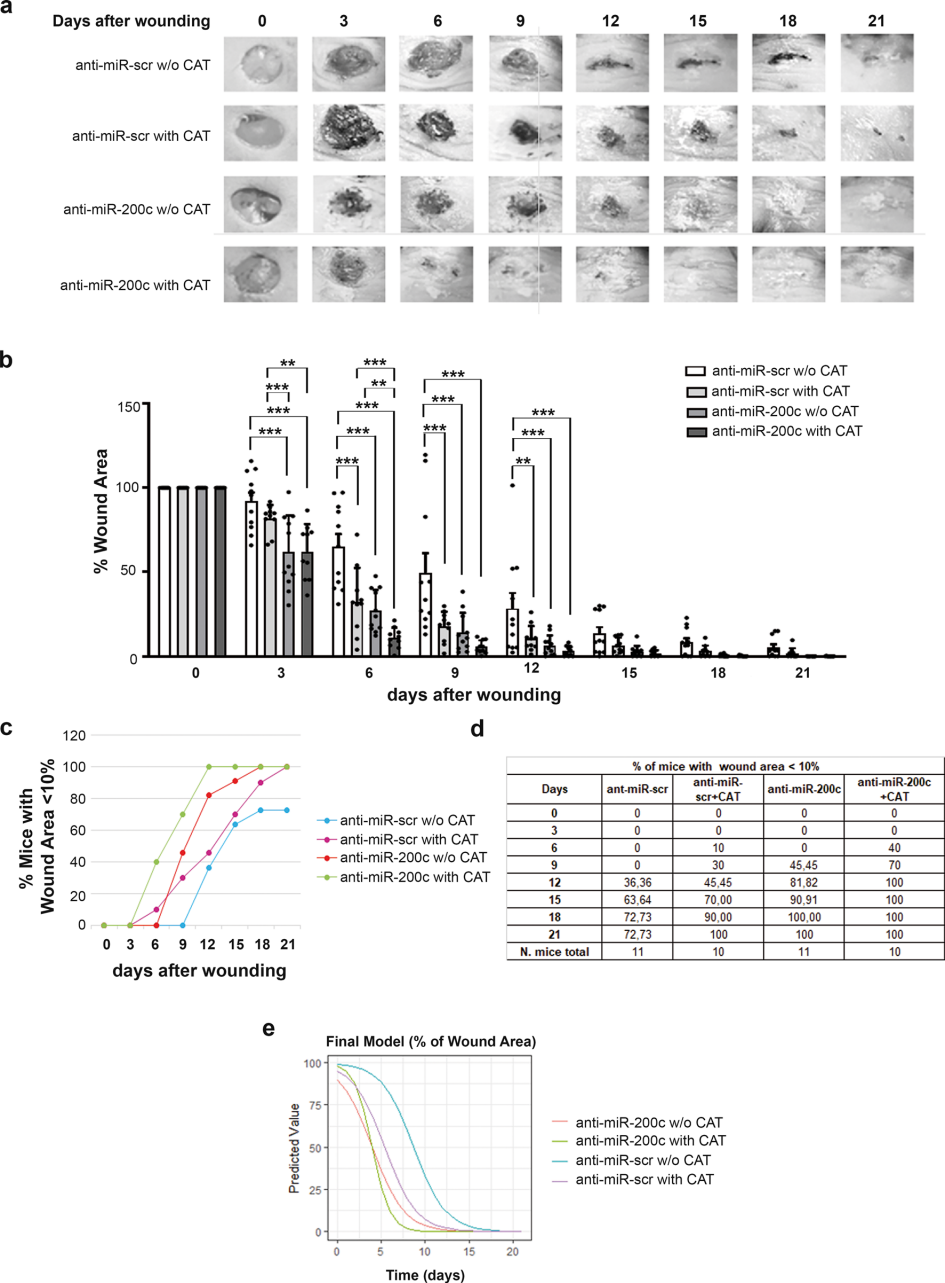
**miR-200c inhibition and CAT treatment accelerates WH in diabetic mice. **Full-thickness excisional wounds of 6 mm were performed on diabetic mice (db/db). The wounds were treated with LNA anti-miR-200c or a control sequence (anti-miR-scr) dissolved in a mixture of 30% Pluronic F-127 gel containing either LNA-scramble or anti-miR-200c (0.04 nmole/μl) in presence or absence of CAT (8UI/μl), every three days until wound closure. **a** The representative panels showed the rate of WH from day 0 to day 21 in the four groups of mice. **b** Bar graph representing the relative wound area expressed in % of wound area *vs* Time 0 for the indicated times and treatments. Two-way Anova, values are expressed as mean ± SEM (N = 11 anti-miR-scr w/o CAT mice, N = 10 anti-miR-scr with CAT mice, N = 11 anti-miR-200c w/o CAT mice, N = 10 anti-miR-200c with CAT mice, *P < 0.05, **P < 0.01, ***P < 0.001; asterisks of statistical significance are *vs* scramble ctrl at each time point, or *vs* the groups indicated by the lines in the bar graphs). **c, d** Graph and table representing the % of mice of different groups that reach a wound area < 10% compared to time zero. 100% of anti-miR-200c + CAT group reached < 10% area at 12 days after wounding, 100% of anti-miR-200c at 18 days, and 100% of CAT group at 21 days. **e** Graphical representation of the decline in the % of WH described by the nonlinear mixed-effects model for the different treatments from the model summary in Table 1

We then examined whether anti-miR-200c and CAT treatment improved WH in a mouse model of diabetes (db/db). After the induction of full-thickness skin wounds, either CAT or LNA-anti-miR-200c alone or together, were topically applied on wounds every three days until wound closure. Anti-miR-200c treatment was effective in decreasing the wound area starting from 3 days after wound and from 6 days for CAT, the co-treatment was effective starting from 3 days (Fig. 10a, b). At day 6 co-treatment was significantly better than anti-miR-200c and CAT treatments alone, and this trend was visible also at further time points (Fig. 10a, b). A percentage of wound area less than 10 compared to time zero was achieved in all mice (100%) for the group of anti-miR-200c + CAT-treated mice at 12 days, for anti-miR-200c-treated group at 18 days, and for CAT-treated group at 21 days after wounding. In the control treated mice (anti-miR-scramble) 8 mice out of 11 (72%) displayed a percentage of wound area less than 10% at 21 days after wounding (Fig. 10c, d).To describe better the decline in the % of WH, a nonlinear mixed-effects model was used for the different treatments (Table 1 and Fig. 10e). The model is parameterized by the rate of decline (scale) and the time to reach 50% of wound area (xmid), allowing for the detection of significant differences among the four groups (the baseline group against which all the groups were compared was anti-miR-200c). This model revealed that xmid for anti-miR-200c and anti-miR-200c + CAT were not different from each other and were significantly different from miR-scramble and CAT treatment alone. The xmid of anti-miR-scramble was 4.75 days later than anti-miR-200c and anti-miR-200c + CAT. Xmid of CAT alone was 1.44 days later than miR-200c or miR-200c + CAT, and 3.31 days before the anti-miR-scramble treatment (Table 1 and Fig. 10e). The rate of decline of different groups, (i.e. the time elapsed between 50 and 75% wound closure), which gives a measure of wound closure speed, shows that anti-miR-200c + CAT declines faster than the other treatments (0.8 less), i.e. anti-miR-200c, anti-miR-scramble and CAT alone treatments, that were not statistically significantly different from each other (Table 1 and Fig. 10e). Indeed, at 21 days wound closure was achieved for anti-miR-200c at 91%, for anti-miR-200c + CAT at 90%, for CAT at 50% and for control at 37%. These results showed that topical treatment with anti-miR-200c and a combination of CAT and anti-miR-200c could be useful treatments to ameliorate WH in diabetic conditions and in ischemic ulcers, than standard procedures with CAT treatment alone [26, 27].

**Table 1:** Nonlinear mixed-effects models

	Estimate	Std. Error	t value	p.val
xmid	3.98	0.39	10.08	< 0.0001***
xmid3	4.75	0.68	7.01	< 0.0001***
xmid4	1.44	0.70	2.10	0.0384*
scal	− 1.83	0.13	− 13.73	< 0.0001***
scal2	0.81	0.27	3.03	0.0024**

xmid comparison among the groups						
	est.diff	se.diff	z.diff	p.val	adjust.p (FDR)	
3–1	4.75	0.68	7.01	2.37 e-12	< 0.0001 ***	(= 3–2)
4–1	1.44	0.70	2.10	3.84 e-02	0.0384 *	(= 4–2)
4–3	− 3.31	0.80	− 4.15	3.29 e-05	< 0.0001 ***	(= 3–2)

xmid3 gives the estimate of the difference in the x-midpoint between anti-miR-scramble (group 3) and groups 1 and 2 (anti-miR-200c w/o CAT and anti-miR-200c + CAT) and xmid 4 gives the estimate of the difference in the x-midpoint between anti-miR-scramble + with CAT (group 4) and groups 1 and 2. Scal2 estimates the difference in the scale parameter between group 2 and groups 1, 3, and 4. anti-miR-200c w/o CAT = 1; anti-miR-200c with CAT = 2; anti-miR-scramble w/o CAT = 3; anti-miR-scramble with CAT = 4

## Discussion

DFU, often leading to repeated lower extremity amputations, is one of the most devastating complications of DM and has a significant socioeconomic impact on the healthcare system [38]. Peripheral artery disease (PAD) is an atherosclerotic occlusive disease of the lower extremity arteries that causes a loss of perfusion, inducing chronic limb-threatening ischemia, characterized by the presence of ischemic rest pain, lower limb ulceration, or gangrene often leading to in lower extremity amputation [43]. Nowadays different treatments are being used for DFU, including revascularization, antibiotics for complicating infections, wound debridement, and off-loading of the ulcer [40]. Various add-on therapies have also been employed, such as hyperbaric oxygen therapy and negative-pressure wound therapy (NPWT) [39]. However, these methods have not demonstrated sufficient efficacy or cost-effectiveness. Although some of these current treatments improve DFU healing, the incidence of amputation and mortality rates remain high. Thus, new, and effective drugs are urgently needed.

ROS are produced by cells under physiologic conditions, and low-level oxidative stress can promote WH [44]. In DFU, there is an imbalance between ROS production and antioxidant enzymes, and excessive oxidative stress has a detrimental effect on different stages of WH, i.e. inflammation, proliferation, and remodelling, leading to endothelial dysfunction, neuropathy, local infection, and ultimately worsening DFU healing [36]**.** In diabetic tissues, excessive ROS production is known to activate PKCβ, which is responsible for p66Shc phosphorylation at Ser-36 [45], further enhancing ROS production [16, 17, 45, 46]. ROS are known to that play a causal role in uncoupling of eNOS [47], a process that leads eNOS to produce superoxide anion (O2−) rather than NO. This process is determined by the bioavailability of the cofactor tetrahydrobiopterin (BH4) [48]. ROS and peroxynitrite (ONOO−), a potent oxidant formed by the reaction of O2− with NO, cause BH4 degradation, leading to eNOS uncoupling. This reduces the amount of NO required for vascular relaxation as well as endothelial cell (EC) survival and proliferation, ultimately causing endothelial dysfunction [47]. The latter is as an abnormal or inappropriate response of the vascular wall to physiological vasodilator stimulus, that is at the basis of the PAD, underpinning chronic ulcers [43].

p66Shc is a stress sensor protein phosphorylated at Ser-36 in response to various ROS-inducing stimuli, including UV-C, H_2_O_2_ in mouse embryonic fibroblasts (FBs) [22], and hyperglycaemic stress [49]. The expression of p66Shc and its phosphorylation at Ser-36 are increased in skin samples from streptozotocin-induced diabetic mice and are also implicated in various diabetes-related complications [50]. Moreover, WH was shown to be significantly faster in p66Shc − / − diabetic mice compared to WT diabetic mice, both with and without hind limb ischemia. Additionally, the ischemic condition further worsens the WH rate compared to diabetes alone [42]. Specifically, it has been shown that in diabetic and ischemic mice, p66Shc deletion accelerates WH by improving re-epithelialization, increasing capillary density, and reducing apoptosis [42]. This is due to the fact that p66Shc-/- mice produce less oxidants and show increased resistance to ROS, reduced ROS-induced apoptosis, and increased resistance to ROS-induced apoptosis [22].

Different antioxidant therapies in animal models and in human studies, have shown a beneficial effect on WH. Among these curcumin, N-acetyl cysteine, quercetin and chitosan are the antioxidant compounds that showed some efficacy, although more research in humans is needed [51]. Another antioxidant enzyme used clinically to promote WH is CAT, is highly expressed in the skin, especially in the stratum corneum, and its activity decreased at the skin surface [52]. This enzyme reacts with hydrogen peroxide producing water and molecular oxygen [53]. Opposite responses were observed with adenovirus-mediated CAT gene transfer and CAT topical application. In a mouse model adenovirus-mediated CAT gene transfer, CAT overexpression impaired WH [54], possibly due to the inhibition of low-level H_2_O_2_ required for vascular endothelial growth factor (VEGF) expression in keratinocytes [54] and for the activation of EGF [55] and keratinocyte growth factor (KGF) receptors [56]. In contrast, topical CAT has been shown to improve WH [23–25], and different commercial products for clinical use contain equine CAT [26, 27].

In this work we have evaluated a novel strategy to prevent redox signalling pathway imbalance, improve skin cell migration, reduce skin cell senescence, apoptosis, and inflammation, and ultimately improve DFU healing.

It has been previously shown that ROS upregulate all miR-200 family members (co-transcribed miR-200c/−141 and co-transcribed miR-200a/−200b/−429) with miR-200c being the most upregulated family member in H_2_O_2_-treated ECs, as well as in human FBs, and in murine myoblasts and myotubes [5–7]. Indeed, there are several lines of evidence to suggest that miR-200c inhibition could be a target to improve DFU healing. miR-200c upregulation causes apoptosis and senescence via ZEB1 target downregulation, and also impairs vascular homeostasis by disrupting the autoregulatory loop among SIRT1, eNOS and FOXO1 [5]. We previously showed that miR-200c promotes ROS production by phosphorylating the stress sensor p66Shc at Ser-36 [5]. Further miR-200c directly targets eNOS, thereby reducing nitric oxide (NO) bioavailability and leading to endothelial dysfunction, a major feature of diabetic vasculopathy [5]. Ischemia, is a pathological condition characterized by ROS increase [57]; our previous studies showed that in a mouse model of hind-limb ischemia miR-200 family is upregulated, and in a p66shc-/- mice, the upregulation was significantly attenuated compared to *wt* mice [7]. Moreover, we found that in hind-limb ischemia muscles, the molecular targets of miR-200c were all decreased, and that the treatment with anti-miR-200c was able to rescue target downmodulation and blood perfusion [5]. Additionally, we previously showed that miR-200c levels were higher in FBs of T2 DB pts compared to HS, and that miR-200c increase correlates with inflammatory cytokines expression of secretory phenotype (SASP), such as IL-6 and monocyte chemoattractant peptide 1 (MCP-1), along with transforming growth factor-beta1 (TGF-B1), suggesting a role for miR-200c in the senescence-associated alterations of DB FBs [58].

In this report, we wanted to dissect the role of miR-200c in the re-epithelialization process of chronic wounds, and to establish the expression levels of its targets SIRT1, FOXO1, ZEB1 and of p66Shc phosphorylation at Ser-36 in delayed DFU healing. We found that miR-200c was significantly induced by different oxidative stress stimuli i.e. H_2_O_2_, HG and UV in immortalized KCs. Furthermore, we observed that miR-200c was significantly increased in biopsies of pts with DFU, and in KCs and FBs derived from the DFU biopsies, compared to biopsies and cells isolated from HS. miR-200c expression in situ was higher in the epidermis compared to dermal layer, and a perinuclear and cytoplasmic localization was observed. Moreover, this technique confirmed higher expression of this miRNA in skin of DFU pts compared to skin of non-diabetic pts undergoing saphenectomies, used as controls. The other miR-200 family members were not significantly modulated in these samples, except for miR-200a and miR-141, that were upregulated only in KCs.

Chronic ulcers are characterized by a stall at the inflammatory phase of wound repair, which contributes to wound closure failure, since there is a dysregulation of macrophage transition from the pro-inflammatory M1 to the anti-inflammatory M2 phenotype [37]. Our results showed that miR-200c was up-regulated in BMDM polarized towards M1 and it was down-regulated in M2. Moreover, we observed a decrease of M1-inflammatory genes, i.e. IL-6, IL-1β, TNF-α and iNOS in anti-miR-200c treated M1 macrophages, demonstrating a role of miR-200c inhibition in decreasing inflammatory pathways and immune cell reprogramming. These results are in line with a previous report in non-alcoholic steatohepatitis (NASH), in which the authors showed that miR-141/200c deficiency diminished inflammation and hepatic steatosis, and that the treatment with LPS of BMDM isolated from miR-200c/141–/– mice polarized macrophages toward the M2 anti-inflammatory state, decreasing the M1 markers [59].

We also analysed miR-200c targets SIRT1, FOXO1 and ZEB1, and we found that they were downregulated at the mRNA and protein levels in skin biopsies and in cells derived from DFU pts. Interestingly, p66Shc mRNA was slightly induced in FBs of DFU pts and was significantly upregulated in KCs and in biopsies of DB; p66Shc protein increased in DB KCs. In line with previous studies that demonstrated p66Shc involvement in diabetes [29], the phosphorylation at Ser-36 of p66Shc was upregulated both in biopsies and in cells derived from DFU pts. This was associated with increased oxidative stress, which we confirmed through DHE fluorescence in diabetic skin biopsies.

SIRT1 is a NAD^+^-dependent deacetylase that plays an important role in the regulation of inflammation and oxidative stress [38]. We previously showed that miR-200c by decreasing SIRT1, induced p53 acetylation, which is known to increase the transcriptional activity of genes implicated in cellular stress, as well as miR-200c itself, which is transcriptionally induced by p53 [5]. Moreover, another SIRT1 target is FOXO1, whose deacetylation activates its transcriptional activity [60]. Therefore, oxidative stress enhances miR-200c, which in turn causes SIRT1 downmodulation, leading to hyper-acetylation of FOXO1 and transcriptional inactivation of its target genes: SIRT1 itself and two important ROS scavengers i.e. MnSOD and CAT [5]. This mechanism enhances oxidative stress, causing phosphorylation of p66Shc at Ser-36, which consequently upregulates ROS in three different cell compartments: the nucleus, mitochondria, and plasma membrane [40, 41].

DM is characterized by an excess of oxidative stress and local inflammation, which are the main cause of apoptosis, inhibition of skin cell proliferation associated with senescence, and failure of cell migration [61]. Combined treatment of CAT and inhibition of miR-200c showed a synergic effect in diminishing oxidative stress*.* Specifically, the co-treatment decreased oxidative stress and restored the expression of most miR-200c targets at mRNA and protein levels in FBs and KCs of DFU pts. Our results show that miR-200c overexpression enhances ROS production in both FBs and KCs, and that the antioxidant CAT decreases ROS. Anti-miR-200c was effective in decreasing p66Shc phosphorylation at Ser-36 in both KCs and FBs of DFU, and the co-treatment further decreased Ser-36-phosphorylation, that reached statistical significance compared to single treatments in KCs and compared to CAT treatment alone in FBs. This indicates that the effect of the combined treatment on oxidative stress is very strong and leads to a decrease in p66Shc Ser-36 phosphorylation, which protects against diabetic-related ischemic damage known to contribute to diabetic foot disease [42]. We also showed that miR-200c overexpression induced cytotoxicity in FBs and KCs of healthy controls and that CAT treatment decreased cytotoxicity of FBs and KCs of DFU pts, and the association with anti-miR-200c further reduced it. In addition, individual treatments with either anti-miR-200c or CAT accelerated WH process in vitro*,* reverting the negative effect caused by two different oxidative stress stimuli, H_2_O_2_ and UV, which both induce miR-200c levels, and the co-treatment showed synergic effect in wound closure. The treatments were also effective in accelerating the rate of WH of primary KCs isolated from diabetic pts undergoing limb amputation, both as single treatments and in combination, with the combination being more effective. We tested the effect of anti-miR-200c and CAT treatment on proliferation in FBs and KCs of DFU pts, but we failed to find an increase in the proliferation rate in these cells, most probably because these cells are senescent, and hence the effect of co-treatment on proliferation is negligible. Thus, the WH effect in vitro is due primarily to the effect on migration induced by different treatments. On the other hand, the overexpression of miR-200c was able to induce proliferation arrest in both FBs and KCs from healthy donors, in keeping with previous studies [62]. We confirmed in vitro results in a mouse model of type 2 diabetes (db/db), which manifests delayed WH. We found that both anti-miR-200c and CAT treatment, when delivered topically on wounds, were effective in ameliorating WH. Furthermore, co-treatment of CAT and anti-miR-200c accelerated the healing process and wound closure more efficaciously than either treatment alone. Interestingly, CAT treatment alone at the beginning was less effective than anti-miR-200c treatment, most likely because decreasing ROS in the initial part of the WH process is not positive, as mentioned above. Anti-miR-200c decreases oxidative stress, but it also modulates other molecular pathways, and the combination of these effects is likely more beneficial than the decrease in ROS alone. Using a logistic model that describes the rate of decline and the time to reach 50% of WH, we found that anti-miR-200c and anti-miR-200c + CAT reached 50% of wound area at a similar time and were significantly different from anti-miR-scramble and CAT treatment alone. The CAT treated group reached 50% wound closure around 1.5 days later than miR-200c or miR-200c + CAT, and ~ 3.5 days before the anti-miR-scramble treatment. The model showed that the rate of decline of different groups from 50 to 75% of wound closure differed among groups and anti-miR-200c + CAT was faster than either treatment alone.

Recent studies showed that not only p66Shc, but also SIRT1 and FOXO1 are involved in chronic WH [41, 63]. Specific deletion of SIRT1 from mouse epidermis has been observed to decrease cell migration, to inhibit tissue regeneration, and to alter the production of inflammatory cytokines [41]. In addition, SIRT1 can protect KCs from genotoxic agent-induced cell death by reducing acetylation of p53 [64]. Therefore, it is tempting to speculate that inhibition of miR-200c, by increasing the expression of SIRT1, could decrease deacetylation of p53, leading to the reduction of its pro-apoptotic activity induced by oxidative stress in diabetic skin, as we already observed in the endothelium [5].

FOXO1 involvement in the WH process is still not clear, but some studies showed that it protects KCs from increased oxidative stress and participated in the inflammatory phase of the WH process, through upregulation of TGF-β1 and its targets, such as integrin-α3, integrin-β6, and metalloproteinases 3 and 9 [63]. Our results showed the decrease of both mRNA and protein of FOXO1 in KCs and FBs of DFU pts and that anti-miR-200c restored their levels. Although the role of FOXO1 is very complex in the WH process, treatment with anti-miR-200c could restore its levels and increase its transcriptional activity, which is useful to transcribe SIRT1 and antioxidant enzymes.

Altogether the results of the present study demonstrate that miR-200c plays an important role in DFU pathogenesis. A prior study investigated the effects of miR-200c on skin WH and KC activity in aging, a condition characterized by increased oxidative stress. It was shown that miR-200c inhibits the migration of KCs, while enhancing their differentiation [62]. Further, miR-200 family is highly expressed in the epidermal layer and all family members target the transcription factors ZEB1 and ZEB2, which act as negative regulators of epithelial-mesenchymal transition (EMT). EMT also occurs in WH and is necessary for KC migration [65]. It has also been shown that in normal skin WH miR-200c expression decreases, and that forced expression of miR-200c represses TGF-β1-induced lamellipodia, stress fiber formation, and intercellular junctional dissolution in keratinocytes [66]. This represents an additional reason useful to demonstrate miR-200 family members may be considered potential candidates for the regulation of re-epithelialization during WH, and that high level of miR-200c can contribute to slow down wound closure. In WH, the coordinated action of pro-inflammatory mechanisms in ECs, FBs, and epithelial cells is closely related to their levels of oxidative stress. The pro-inflammatory role of miR-200c has been studied mainly at the vascular level in diabetic conditions. Indeed, it was observed that in a diabetic mouse model, vascular smooth muscle cells exhibit elevated levels of some members of the miR-200 family, including miR-200c [11]. In this study, it was also observed that the decrease in ZEB1 induced the expression of different pro-inflammatory genes [11]. In a recent paper it was shown that in diabetic animals in the delayed healing wounds the reprogramming of FBs into endothelial-like cells was blunted, and that topical tissue delivery by nano-electroporation of anti-miR-200b oligonucleotide, another member of miR-200 family that share the same seed sequence of miR-200c, was sufficient to restore vasculogenic fibroblast emergence, tissue perfusion, and WH [67]. Therefore, these results are in line with our previous data showing a beneficial effect of anti-miR-200c on limb perfusion in mice upon ischemia [5], and suggest a possible contemporary inhibition of miR-200b and miR-200c.

The results of the present work showed that miR-200c induced oxidative stress in FBs and KCs of DFU pts, through a mechanism involving p66Shc phosphorylation at Ser-36 and decreased expression of its molecular targets: SIRT1 and the transcription factors ZEB1 and FOXO1.

Since the WH process depends on a proper balance of redox state, miR-200c upregulation in pts with DFU contributes to slow down the diabetic wound closure process. Treatment with anti-miR-200c could act on multiple molecular targets simultaneously by decreasing the inflammatory state, oxidative stress, apoptosis and skin and vascular cell senescence. Anti-miR-200c when acting on the vascular district, could improve blood perfusion, restore vascular function, and reverse all the negative effects caused by diabetic and ischemic conditions. In the skin district, it accelerates cell migration, thereby improving the healing process.

**Fig.11:**
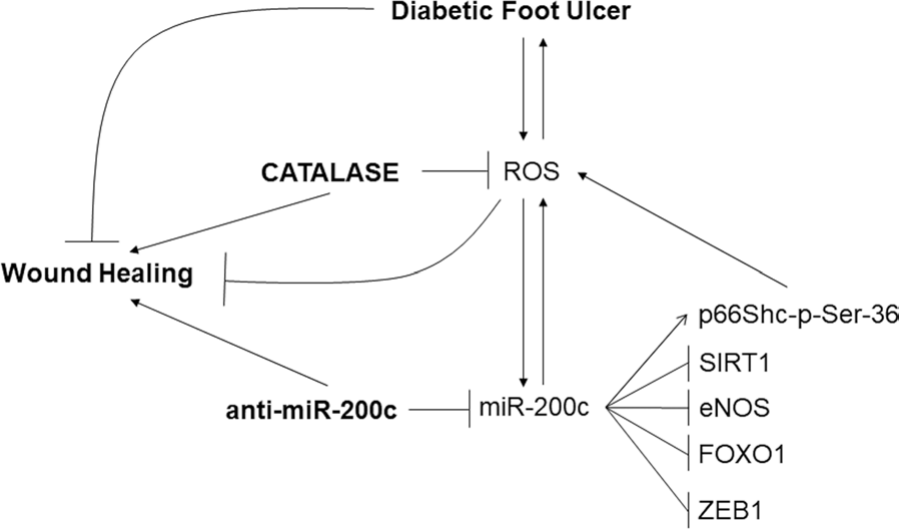
**Proposed mechanism of action of miR-200c inhibition and CAT treatment in DFU healing.** DFU displays an increased level of oxidative stress caused both by hyperglycaemia and ischemia; ROS, further enhance miR-200c expression that, in turn, worsens oxidative stress and delays DFU healing. miR-200c downregulates its direct targets ZEB1, SIRT1, eNOS and FOXO1, and also induces the phosphorylation of p66Shc at Ser-36, which upregulates ROS. Anti-miR-200c and CAT decrease oxidative stress, inflammation, skin and vascular cell senescence and death and, by these mechanisms promote DFU healing. Thus, topical administration of a combination of anti-miR-200c and CAT holds promise as a novel therapeutic approach for chronic DFU

Interestingly, we found that the simultaneous treatment with anti-miR-200c and CAT decreased further miR-200c levels both in FBs and KC of DFU pts compared to single treatments (data not shown), further contributing to the WH process. These results demonstrated that the final effect of the anti-miR-200c and CAT treatment is the downregulation of oxidative stress in two different ways: exogenously (by CAT enzyme) and endogenously (by anti-miR-200c), which work in synergy, ultimately leading to a decrease in miR-200c and oxidative stress, downregulating inflammation, apoptosis, skin and vascular cell senescence, and increasing migration, thereby accelerating WH (Fig. 11).

## Conclusions

Taken together, the results of the present study suggest that miR-200c inhibition in conjunction with topical CAT treatment could represent a novel strategy for DFU treatment and, possibly, for other types of non-diabetic skin ulcers.

## Supplementary Information

Additional file 1

Additional file 2

## Data Availability

The datasets supporting conclusions of this article are available from the corresponding author upon reasonable request.

## Abbreviations

BMDM: Bone marrow-derived macrophages
CAT: Catalase
DHE: Dihydroethidium
DFU: Diabetic foot ulcers
ECs: Endothelial cells
EGF: Epidermal growth factor
eNOS: Endothelial nitric oxide synthetase
FBs: Fibroblasts
FOXO1: Forkhead box protein O1
h: Hours
HG: High glucose
H_2_O_2_: Hydrogen peroxide
IHC: Immunohistochemistry
IL: Interleukin
iNOS: Inducible nitric oxide synthase
ISH: In situ hybridization
KCs: Keratinocytes
KGF: Keratinocyte growth factor
MCP-1: Monocyte chemoattractant peptide 1
miRNA: MicroRNA
MnSOD: Manganese superoxide dismutase
mRNA: Messenger RNA
NO: Nitric oxide
NPWT: Negative pressure wound therapy
ONOO −: Peroxynitrite
Pts: Patients
p66Shc P-Ser36: Phosphorylation at Serine 36 of p66Shc protein
ROS: Reactive oxygen species
SIRT1: Sirtuin 1
SASP: Senescence-associated secretory phenotype
TNFα: Tumor necrosis factor-alpha
O2 −: Superoxide anion
TGF-B1: Transforming growth factor-beta1
UV: Ultraviolet radiations
WH: Wound healing
ZEB1: Zinc finger E-box binding homeobox 1
