# Supplementary material for: miR-200c inhibition and catalase accelerate diabetic wound healing
Source: J Biomed Sci. 2025 Feb 14;32:21. doi: 10.1186/s12929-024-01113-7 (PMC11827459; doi:10.1186/s12929-024-01113-7)
Supplement: Supplementary file 1 — Additional file 1 [file 12929_2024_1113_MOESM1_ESM.docx]

**Supplementary Figures and Legends**

**Supplementary Fig.1**


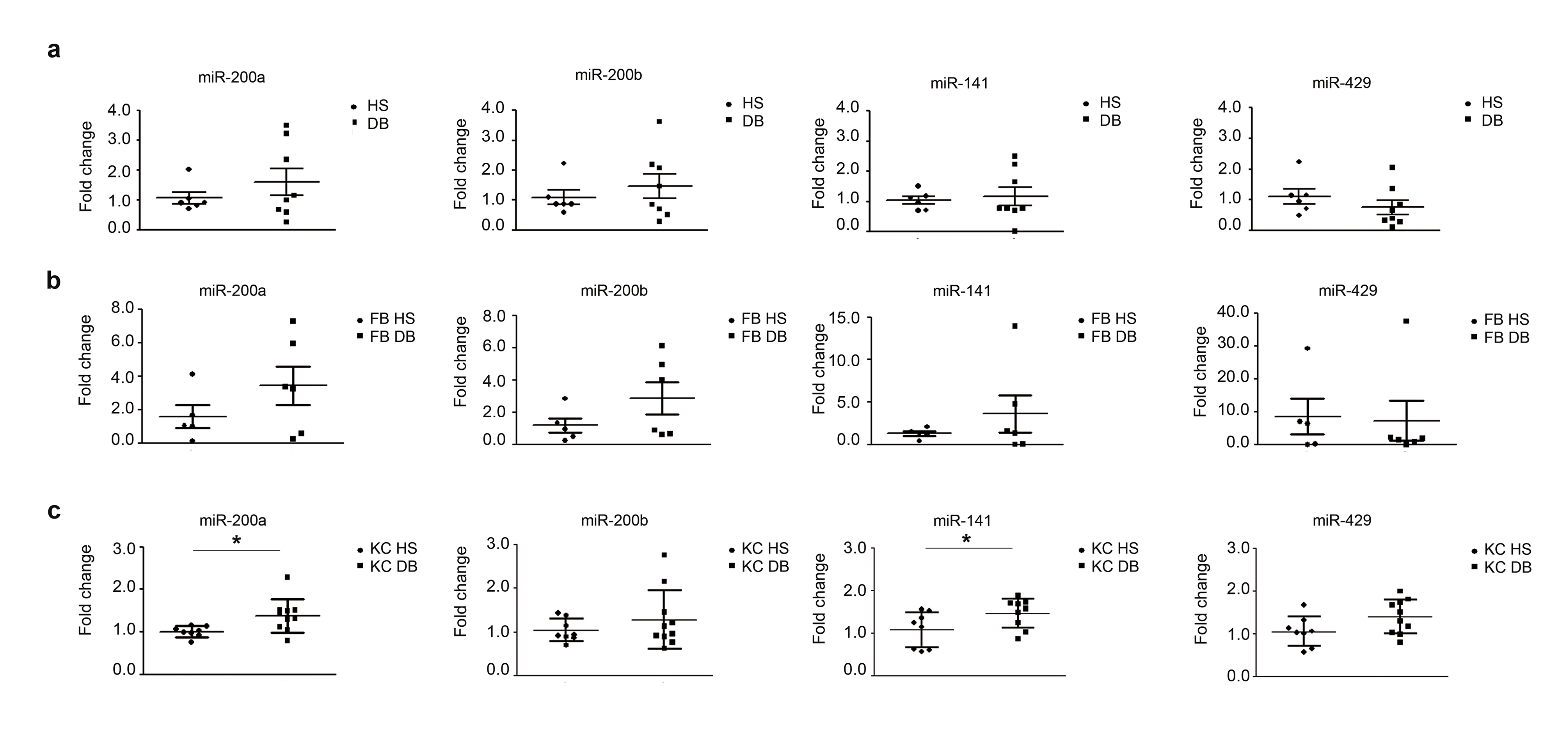


**Supplementary Fig.1. miR-200 family modulation in skin biopsies, FBs, and KCs of diabetic foot ulcers patients.** a) miR-200 family expression in human skin biopsies of DFU pts (DB) compared to HS pts (N=5 HS, N=8 DB). b) miR-200 family members were evaluated in RNA extracted from FBs isolated from skin biopsies of patients with DFU (FB DB) or HS skin biopsies (FB HS) (N=5 HS, N=6 DB). c) miR-200 family members were evaluated in RNA extracted from KCs isolated from skin biopsies of DB (KC DB) or HS (KC HS) skin biopsies (N=8 HS, N=8 DB; *p<0.05).

**Supplementary Fig.2**





**Supplementary Fig. 2. miR-200c expression in cells transduced with miR-200c or anti-miR-200c.** Primary FBs and KCs derived from skin biopsies of HS were transduced with a lentivirus encoding miR-200c or with a control virus (miR-scr). a) miR-200c expression was evaluated in RNA extracted from FBs transduced with miR-200c (N=6; *p<0.05). b) miR-200c expression was evaluated in RNA extracted from KCs transduced with miR-200c (N=6; *p<0.05). c,d,e) FBs and KCs of DFU pts and HaCaT were infected either with a lentivirus encoding anti-miR-200c or with a control virus. After 24h, cells were selected with puromycin and after selection total RNA was extracted (N=6; *p<0.05).

**Supplementary Fig.3**





**Supplementary Fig. 3. miR-200 family modulation in FBs and KCs of diabetic foot ulcers patients upon CAT treatment.** a) miR-200 family member expression was evaluated in RNA extracted from FBs of DFU pts treated with equine 400UI/ml CAT for 16h (N=6 FB DB). b) miR-200 family member expression was evaluated in RNA extracted from DFU KCs treated with CAT for 8h (N=6 KC DB).

**Supplementary Fig.4**


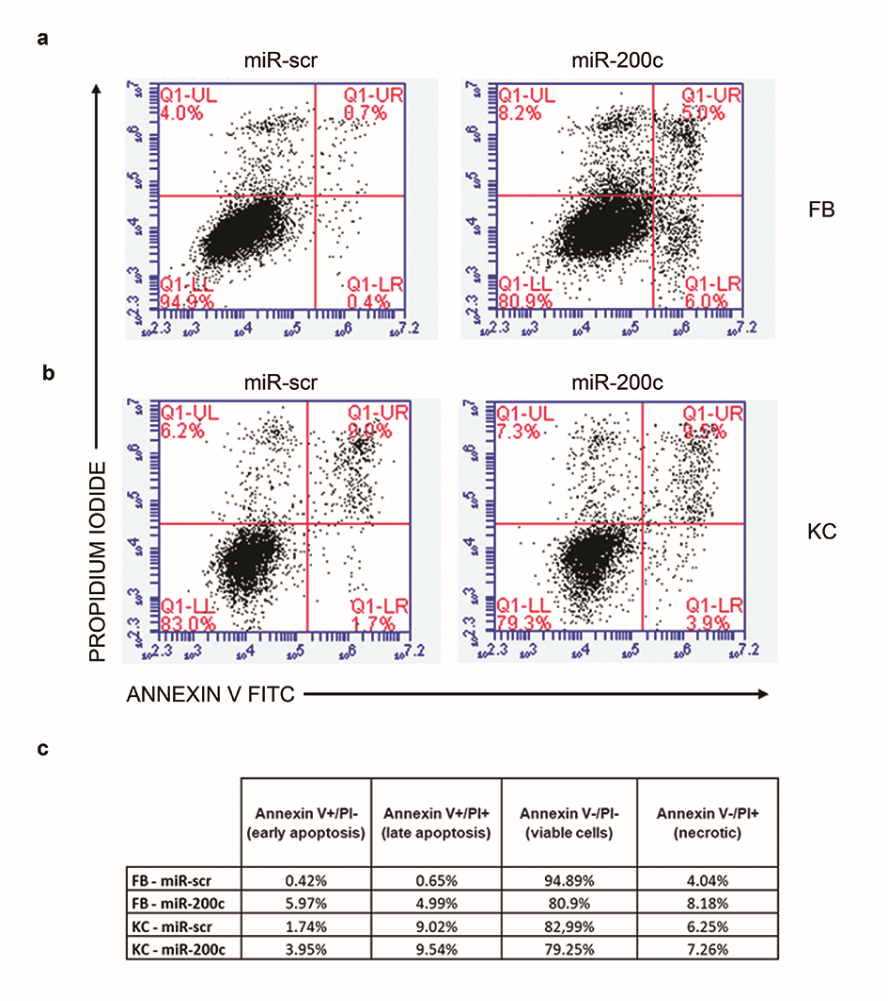


**Supplementary Fig. 4.** **miR-200c reduces the viability and induces apoptosis in FBs and**

**KCs of HS**. a-b) FBs and KCs of HS were infected either with a lentivirus encoding miR-200c or a miR-scr control. After 16h, apoptosis was evaluated by measuring Annexin/PI fluorescent staining through FACS analysis. c) Table showing the percentage of Annexin V/PI fluorescence staining.

**Supplementary Fig.5**


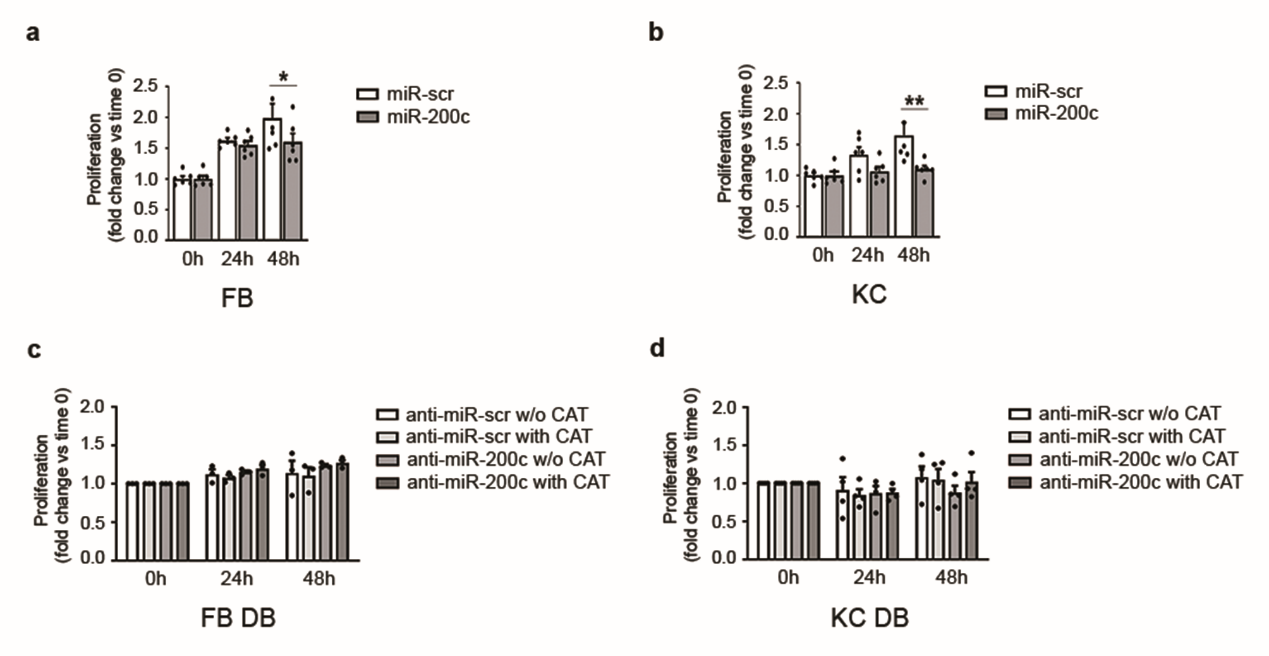


**Supplementary Fig. 5. miR-200c and catalase effect on proliferation.** a-b) Primary FBs and KCs derived from skin biopsies of HS were transduced with a lentivirus encoding miR-200c or with a control virus (miR-scr). Cell proliferation was assayed at the indicated times. miR-200c decreased proliferation at 48h in both FBs and KCs (N=6; *p<0.05; **p<0.01). b) FBs and KCs of DFU pts were infected either with a lentivirus encoding anti-miR-200c or with a control virus (anti-miR-scr). Then cells were incubated with 400 UI/ml equine CAT and proliferation was assayed at the indicated times. Proliferation was not affected by either single treatments or co-treatment with anti-miR-200c or CAT.

**Supplementary Fig.6**





**Supplementary Fig. 6**. Uncropped Western Blot for figure 4 and 7.
