# Supplementary material for: miR-200c inhibition and catalase accelerate diabetic wound healing
Source: J Biomed Sci. 2025 Feb 14;32:21. doi: 10.1186/s12929-024-01113-7 (PMC11827459; doi:10.1186/s12929-024-01113-7)
Supplement: Supplementary file 2 — Additional file 2 [file 12929_2024_1113_MOESM2_ESM.docx]

**Supplementary Table 1**

**Clinical and laboratory parametrs of DFU pts**

| **SEX** | **Age** | **HbA1c**  **(mmol/mol)** | **Fasting glucose**  **(mg/dl)** | **Duration**  **of diabetes**  **(>10 Years)** |
| --- | --- | --- | --- | --- |
| M | 83 | 44 (6.2%) | 130 | yes |
| F | 72 | 64 (8.0%) | 188 | yes |
| M | 81 | 54 (7.1%) | 177 | yes |
| M | 73 | 56 (7.3%) | 169 | yes |
| F | 94 | 58 (7.5%) | 171 | yes |
| M | 84 | 47 (6.5%) | 130 | yes |
| M | 85 | 49 (6.6%) | 140 | yes |
| M | 70 | 45 (6.3%) | 140 | yes |
| M | 45 | 43 (6.1%) | 119 | no |
| F | 53 | 43 (6.1%) | 107 | no |
